# Precise heteroatom doping determines aqueous solubility and self-assembly behaviors for polycyclic aromatic skeletons

**DOI:** 10.1038/s42004-022-00724-1

**Published:** 2022-08-29

**Authors:** Kang Li, Jia-Min Hu, Wei-Min Qin, Jing Guo, Yue-Peng Cai

**Affiliations:** 1 School of Chemistry, South China Normal University, 510006 Guangzhou, China; 2 School of Chemistry, Sun Yat-Sen University, 510275 Guangzhou, China

**Keywords:** Chemical physics, Nanoscale materials, Self-assembly, Materials chemistry

## Abstract

Developing effective strategies to improve the hydrophilicity or aqueous solubility of hydrophobic molecular scaffolds is meaningful for both academic research and industrial applications. Herein, we demonstrate that stepwise and precise N/O heteroatoms doping on a polycyclic aromatic skeleton can gradually alter these structures from hydrophobic to hydrophilic, even resulting in excellent aqueous solubility. The Hansen solubility parameters (HSP) method shows that the three partial solubility parameters are closely related to N/O doping species, numbers and positions on the molecular panel. The hydrogen bonding solubility parameter indicates that the hydrogen bonding interactions between N/O doped molecules and water play a key role in enhancing hydrophilicity. Moreover, three optimized water-soluble molecules underwent a self-assembly process to form stable nanoparticles in water, thus facilitating better hydrogen bonding interactions disclosed by HSP calculations, NMR and single crystal X-ray analysis. These ensembles even show quasi-solid properties in water from NMR and luminescence perspectives.

## Abstract (web summary)

Enhancing the hydrophilicity of hydrophobic molecular scaffolds allows to increase their aqueous solubility and therefore their usability for a range of applications. Here, N/O heteroatom doping of polycyclic aromatic hydrocarbons is shown to switch the skeleton from hydrophobic to hydrophilic, enhancing aqueous solubility and facilitating self-assembly in water.

## Introduction

The term “solubility” is a basic concept for substances to describe their compatibility with specific solvents and defined as the maximum amount of solute that can dissolve in a given amount of solvent at a certain temperature^1^. This property is significant in the field of pharmacy^2,3^, pigment^4^, environmental predictions^5,6^, agrochemical design^7^, protein folding^8,9^, and so on. In most cases, the simple and empirical rule “like dissolves like” can direct us to qualitatively estimate the compatibility between solutes and solvents^10^, which means substances with similar chemical characteristics will dissolve in each other.

Among these, aqueous solubility is of fundamental interest to both academia and industry^11,12^. This is mainly attributed to the following reasons: (1) water is the most conveniently available solvent; (2) green chemistry concern; (3) the characteristic hydrophobic effect; (4) water constitutes the basis of media for a biological system. However, most existing chemicals have undesirable compatibility with water and many approaches have been developed to enhance the hydrophilicity or aqueous solubility of specific substances^2^. For instance, the cosolvent method that adding an organic solvent to the aqueous solution is one of the most common and effective ways for solubility enhancement^13^; the formation of hydrochloride as salt for targeted substance is widely applicable in pharmaceuticals to fulfill biological compatibility^14^; the surfactant or host structures (e.g., cyclodextrin, capsule, coordination cage) as hydrotropic agent can transfer hydrophobic substances to water phase through forming inclusion complex^12,15–18^. In addition, chemists can use their synthetic toolbox to modify molecules with functional groups to achieve aqueous solubility enhancement purpose (e.g., decorating hydrophilic polyethylene glycol long-chain on molecular skeleton)^19^.

**Fig. 1:**
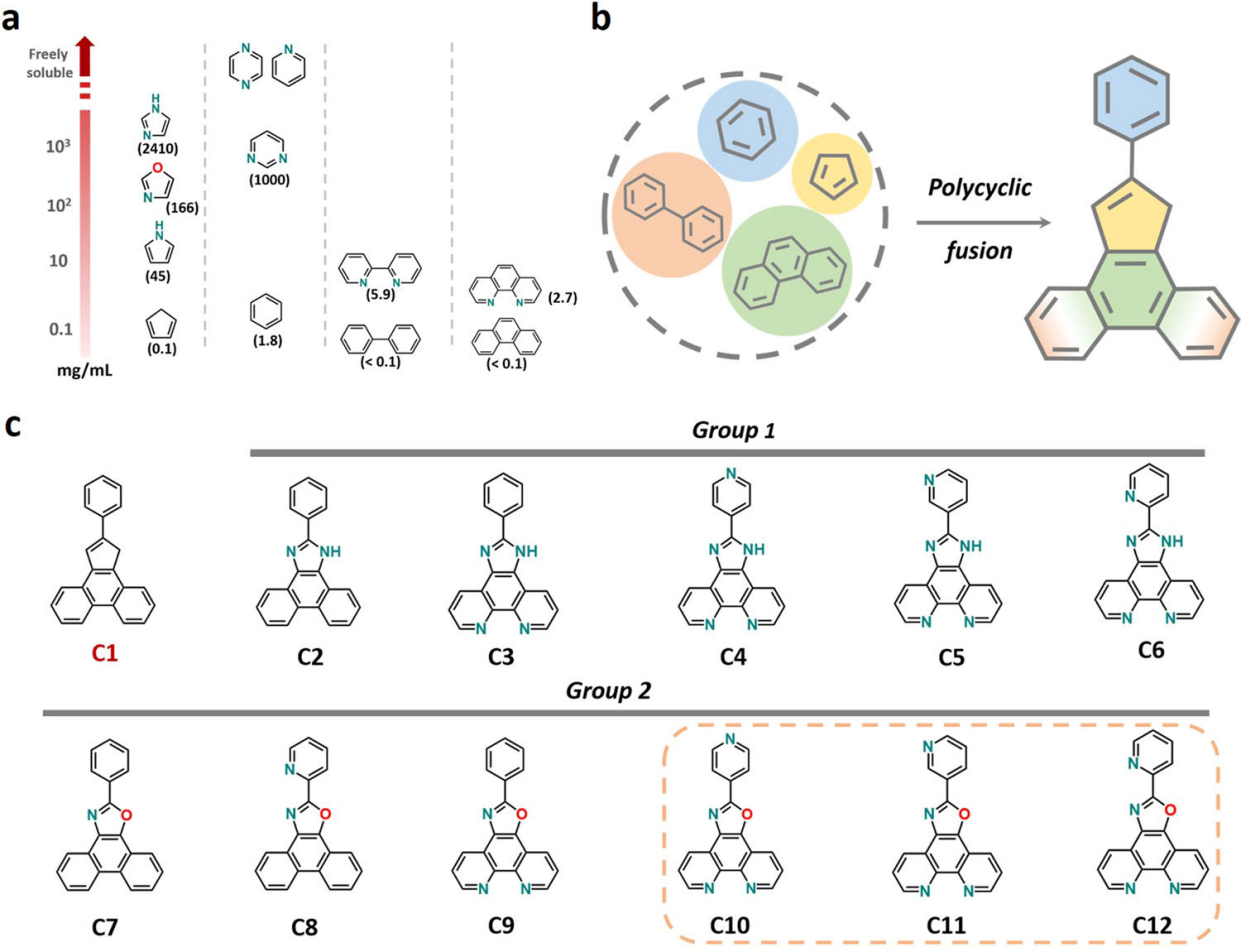
The evolution of N/O doping from simplicity to complexity. **a** Aqueous solubility of simply common aromatic hydrocarbons and their corresponding N/O doping analogs at 25 °C (data in parentheses was obtained from hazardous substances data bank). **b** Molecular structure design via diversified aromatic polycycles fusion. **c** Combination of programmed N/O doping towards pristine **C1** structure (highlighted molecules demonstrate excellent aqueous solubility).

Polycyclic aromatic hydrocarbons (PAH) widely exist in chemical libraries and found applications in sensing, luminescence, electrochemistry, material science, etc. ^20–23^. Moreover, the B/N heteroatoms are often doped to PAH skeleton to tune their electronic structures and related physicochemical properties^24–26^, seldom focusing on the influence of aqueous solubility considering most PAH are superhydrophobic and insoluble in water due to their nonpolar and lipophilic nature^27^. Nevertheless, the subcomponents of PAH with N/O doping show entirely different hydrophilic performances. As shown in Fig. 1a, compared with benzene possessing poor aqueous solubility, the corresponding N-doped pyridine and pyrazine are freely soluble in water. N/O heteroatoms doping to the cyclopentadiene skeleton also greatly enhance the aqueous solubility. For medium-sized molecules (diphenyl, phenanthrene), we can still observe an extent of aqueous solubility improvement derived from the doped N atom on the panels. Along this line, we intend to explore this trend and propose a useful strategy that properly doping N/O heteroatoms on the polycyclic aromatic skeletons with relatively large sizes may greatly improve their hydrophilicity and aqueous solubility.

**Fig. 2:**
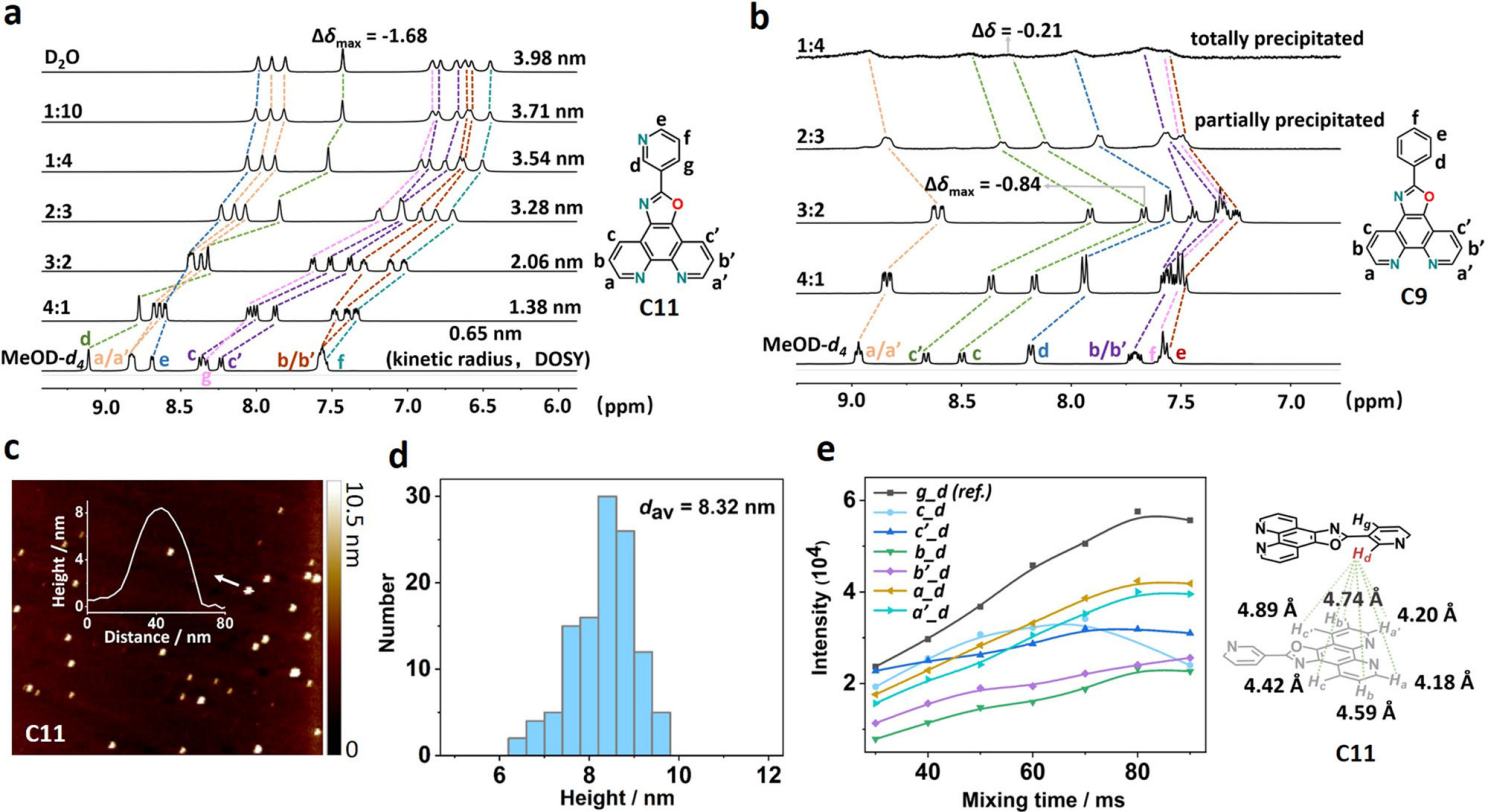
Investigation of self-assembly behaviors in aqueous solution. **a** Stacked ^1^H NMR spectra of **C11** in MeOD-*d*_4_/D_2_O mixture with variable volume ratios (each proton was assigned and labeled along with different solvent ratios, the kinetic radii of ensembles under each condition were measured by DOSY spectra and calculated by Stokes-Einstein equation). **b** Stacked ^1^H NMR spectra of analogous **C9** in MeOD-*d*_4_/D_2_O mixture with variable volume ratios. **c**, AFM analysis of **C11** in aqueous condition (a height profile of selected nanoparticle was inserted). **d** Statistical distribution of **C11**-based nanoparticle number versus size. **e** Left: NOE build-up curves for **C11** in D_2_O, right: calculated intermolecular adjacent proton distances with peak d derived from the NOE growth rate analysis using peak g-d correlation as internal reference.

To verify our hypothesis, we chose a polycyclic aromatic skeleton (**C1**: 2-phenyl-1*H*-cyclophen[*l*]phenanthrene^28^) with more complexity as modeling compound (Fig. 1b). Two main reasons lead to this selection: (1) **C1** fuses with 5-membered, 6-membered and condensed aromatic ring on its skeleton, which seems like a molecular splicing process using all subcomponents summarized in Fig. 1a; (2) ease to synthesize N/O doped analogs with only one or two-steps workup procedure. Eventually, 12 combinatorial molecules (**C2**-**C12**) with variable N/O doping numbers and positions were designed and successfully synthesized (presented in Fig. 1c). Compared with pristine **C1**, the hydrophilicity of these derived molecules is mostly improved. To our delight, three molecules with optimized N/O doping show excellent aqueous solubility (**C10**-**C12**). Furthermore, these water-soluble molecules underwent an aggregation process and form nanoparticles in aqueous media to facilitate the hydrogen bonding interactions, which results in a series of unexpected phenomena including NMR signals change, luminescence shift, and others. These results greatly support our anticipation at the beginning for hydrophilic properties improvement purposes.

## Results and discussion

### Synthesis and solubility parameters

We have employed various diones and aldehydes as starting materials to successfully obtain the desired N/O-doped structures as demonstrated in Fig. 1c. These molecules can be classified into two groups with solely N doping (group 1) and dual N/O doping (group 2). The one-step procedure of condensation reactions between diones and aldehydes in acetic acid directly gave rise to **C2**-**C6**. For **C7**-**C12**, an additional step of reducing one ketone to imine under ammonia gas atmosphere was required. Thus, the half-reduced intermediate can react with aldehydes to give the target molecules. These synthesized molecules have been fully characterized by ^1^H/^13^C NMR spectroscopy and HRESI-MS spectrometry (Supplementary Methods 1.1 and 1.2).

With the 12 combinatorial molecules in hand, we initially examined their hydrophilicity and aqueous solubility. The water-contact angle test gave a clear hydrophilic assessment for these molecules (Table 1 and Supplementary Table 1). The conventional shake-flask method was employed to obtain the thermodynamic aqueous solubility data^29^. Although we did not synthesize the model molecule **C1**, the nonpolar aromatic hydrocarbon backbone reflects its superhydrophobic nature and neglectable aqueous solubility. In group 1, **C2** holding two N atoms on the imidazole subcomponent seems to remain hydrophobicity with the water-contact angle of 140.6°. When two more N atoms are introduced to the phenanthrene panel, **C3** (0°) displays a jump from hydrophobicity to hydrophilicity compared with **C1** and **C2**. It’s reasonable that the following **C4**-**C6** with another N atom doping on different positions of terminal benzene ring share the same water contact angle of 0°. It is obvious that with the stepwise doping N atom to different parts of **C1**, these derived molecules demonstrate improved hydrophilicity. In contrast, **C2**-**C6** all show relatively low aqueous solubility (<0.1 mg/mL), and little improvement is observed as we expected. In pursuit of both improving the hydrophilicity and aqueous solubility, we planned to introduce dual N/O heteroatoms to **C1** skeleton as listed in group 2. Initially, when doping N/O atoms to two sides of cyclopentadiene subcomponent to form oxazole ring, **C7** remains high hydrophobicity with a 135.2° water contact angle. Additional doping N atom on the terminal benzene ring for **C8** slightly reduces the angle to 128.6°, yet is hydrophobic as well. The turning point comes to **C9** that doping two N atoms on the phenanthrene subcomponent besides the oxazole part. A water contact angle of 0° indicates the high hydrophilicity of **C9**. Consequently, the following compounds **C10**-**C12** with more N atom doping on the terminal benzene ring are also highly hydrophilic. The aqueous solubility for group 2 molecules showed distinct results that are different from group 1. **C7** and **C8** are barely soluble in water due to their high hydrophobicity. **C9** is slightly soluble that implies an improving trend. Inspiringly, **C10**-**C12** display good to excellent aqueous solubility with a maximum of 150 mg/mL (**C12**).

**Table 1:** Solubility-related parameters of **C1**–**C12**.

No.	Water contact angle (°)	Aqueous solubility (mg/mL)	*δ*_D_ (MPa) ^0.5^	*δ*_P_ (MPa) ^0.5^	*δ*_H_ (MPa) ^0.5^	*R*_0_	Water1^a^		Water2^a^	
							*R*_a_	RED^b^	*R*_a_	RED^b^
C1^c^	>140	<0.1	17.8	1.5	3.1	3.0	23.8	8.0	20.8	6.9
C2	140.6	<0.1	18.5	12.6	8.1	4.5	13.3	3.0	9.9	2.2
C3	0	<0.1	17.9	9.1	10.1	5.4	14.1	2.6	10.5	1.9
C4	0	<0.1	16.4	15.6	16.1	7.5	5.5	0.7	3.8	0.5
C5	0	<0.1	16.4	15.8	16.5	7.0	5.3	0.8	3.7	0.5
C6	0	<0.1	16.1	12.8	16.8	7.9	7.9	1.0	5.9	0.7
C7	135.2	<0.1	19.5	7.2	7.5	6.6	18.2	2.8	13.9	2.1
C8	128.6	<0.1	19.4	7.0	7.8	8.8	18.1	2.1	13.8	1.6
C9	0	0.9	18.6	12.3	15.5	10.0	10.8	1.1	5.1	0.5
C10	0	40.0	17.7	6.3	15.8	10.7	15.0	1.4	10.8	1.0
C11	0	13.0	18.3	7.0	15.9	11.0	14.9	1.4	10.2	0.9
C12	0	150.0	19.4	6.5	17.2	12.3	16.4	1.3	10.9	0.9

^a^Water1 refers to the water HSP of dilute condition as (15.1, 20.4, 16.5), while Water2 refers to the water HSP of dense condition as (18.1, 17.1, 16.9), the corresponding calculations of *R*_a_ and RED are based on the two conditions, respectively.

^b^RED is short of relative energy difference and introduced as criteria to assess the affinities and defined as the ratio of *R*_a_ and *R*_0 _(*R*_a_/*R*_0_). Obviously, three conditions are classified: (1) RED < 1.0 indicates high affinity; (2) RED ≈ 1.0 is a boundary condition; (3) RED » 1.0 indicates low affinity.

^c^The water contact angle and aqueous solubility of **C1** were deduced and estimated when compared with **C2**, its HSP was calculated from a distinct solvent library (see supplementary information).

To figure out why the doped N/O heteroatoms on the aromatic skeletons can both influence their hydrophilicity and aqueous solubility, we employed the Hansen solubility parameters (HSP) to interpret this phenomenon. This method developed by Charles Hansen et al.^30^ has found broad use both in academia and industry for predicting the compatibility or affinity between two substances^31–34^. The key elements of HSP approach are three partial solubility parameters consisting of *δ*_D_, *δ*_P,_ and *δ*_H_, in which *δ*_D_ represents the dispersion solubility parameter, *δ*_P_ represents the polar solubility parameter, and *δ*_H_ represents the hydrogen bonding solubility parameter, respectively.

The three partial parameters define a three-dimensional coordinate for substances in a virtual solubility space (Hanse space). In principle, the closer the two coordinates of HSP are, the more likely the substances will dissolve in each other, which is consistent with the notion of “like dissolves like” rule. To better describe the degree of closeness, another solubility parameter “distance” (*R*_a_) is introduced as follows:

1 $${R}_{{\rm {a}}}^{2}=4{({\delta }_{{\rm {D}}2}-{\delta }_{{\rm {D}}1})}^{2}+{({\delta }_{{\rm {P}}2}-{\delta }_{{\rm {P}}1})}^{2}+{({\delta }_{{\rm {H}}2}-{\delta }_{{\rm {H}}1})}^{2}$$

Meanwhile, each substance has an intrinsic distance parameter *R*_0_, together with HSP as a center point that would define a solubility sphere in Hansen space. If the HSP coordinate of one substance is located inside the sphere of another substance, it indicates a high affinity between them. For solute and solvent cases, a dissolution process may happen. Otherwise, a low affinity is inferred if one substance is excluded from the sphere of another.

The total HSP data set of **C1**-**C12** was experimentally tested and optimized from a combination of solvents library and listed in Table 1 (Supplementary Methods 1.3, Supplementary Fig. 1). As is seen, the calculated intrinsic distance parameter *R*_0_ is gradually increased along with the extent of N/O doping from **C1** to **C12**, indicative of improved compatibility with more organic solvents. For the unique H_2_O, there are three sets of HSP for different conditions. The first is the HSP of single-molecule (pure) water (15.5, 16.0, 42.3), in which the *δ*_H_ is more salient than *δ*_D_ and *δ*_P_, it well explains the strong hydrogen bonding interactions between water molecules. However, its use in the prediction of solubility in water is not deemed appropriate in most cases. The second and third sets of HSP for water are those derived from solubility data and should be chosen case by case. The second set of HSP (15.1, 20.4, 16.5) was derived from experimental data set exceeding 1% soluble compounds in water, which may be appropriate for the diluted solute case (denoted as Water1). The third set of HSP (18.1, 17.1, 16.9) was derived from experimental data set of complete miscible compounds with water, which may be appropriate for the dense solute case (denoted as Water2).

In practice, Water1 and Water2 were both used for **C1–C12** to fulfill calculations for comparison. For the group of only N doping (**C1–C6**), although the distances between designed molecules and H_2_O (*R*_a_) are considerably reduced, both HSP conditions give the criterion parameter RED >1.0 as the boundary for **C1–C3**, which is consistent with the experimental water solubility testing results; while Water1 and Water2 give relatively smaller RED below 1.0 for **C4–C6**, especially under Water2 condition which even generates a low value of 0.5 for **C4–C5**. Considering the bad water solubility of **C4–C6** from experimental results, the derived *R*_a_ and RED parameters may be underestimated under Water2 condition. However, the Water1-based analysis still gives a paradoxical prediction of good water compatibility versus the bad experimental water solubility performance, which may be attributed to the complexity of solubility issues, especially in water conditions. For the group of dual N/O doping (**C7–C12**), Water1 and Water2-based analysis give distinct results in comparison. Under the Water1 condition, all the REDs exceed the boundary value of 1.0, indicative of bad compatibility with water. By contrast, under the Water2 condition, the REDs of **C9–C12** are equal or below 1.0 while **C7** and **C8** still exceed the boundary value that is consistent with the Water1 result. As mentioned above, the two sets of water HSP should be chosen case by case for specific analysis. Considering **C7–C9** with bad/poor aqueous solubility and **C10–C12** with good to excellent aqueous solubility from the perspective of experimental results, the Water1 parameters shall be chosen for **C7–C9** as a dilute solute case, while the Water2 parameters shall be chosen for **C10–C12** for the dense solute case. Under this guiding principle, the calculation results of water solvent excluded from **C7–C9** solubility sphere and included in **C10–C12** solubility sphere using RED as an indicator match well with their experimental aqueous solubility performance. It is noteworthy that although **C10–C12** could include H_2_O in their solubility sphere, the corresponding REDs (0.9–1.0) are still near the boundary condition and should be treated carefully. The following investigations of molecular behaviors in solution and intermolecular hydrogen bonding may provide certain mechanistic insight into the remarkable aqueous solubility performance.

### Self-assembly behaviors in aqueous solution

Alternatively, we employed NMR technology to probe the existing state of soluble molecules **C10–C12** in aqueous solution. Taking **C11** for instance (Fig. 2a), with the increase of water content in MeOD-*d*_4_/D_2_O mixture, all the signals moved upfield and the overlapped peaks split into discrete ones. Finally, 10 distinguishable single peaks appeared corresponding to 10 inequivalent protons on **C11** skeleton in pure D_2_O. The major upfield chemical shifts exceeded 1.00 ppm with a maximum of 1.68 ppm for peak d. These results suggested a strong chemical shielding effect between adjacent aromatic panels in close proximity, which may be caused by molecular aggregation in aqueous solution. Furthermore, the measured kinetic radii from DOSY testing showed a significant increase from 0.65 nm in MeOD-*d*_4_ to 3.98 nm in D_2_O, which again confirmed our anticipation that an aggregation process gradually occurs with the increase of water content (Supplementary Figs. 5, 8 and 11). Similar phenomena also happen for **C10** and **C12** (Supplementary Figs. 2–4, 6, 7, 9, 10 and 12). In comparison, we selected **C9** with relatively poor aqueous solubility (0.9 mg/mL) to demonstrate the changing process under the same operation (Fig. 2b). When aliquots of D_2_O were introduced to **C9** in MeOD-*d*_4_, all the signals uniformly moved to upfield until the 3:2 ratio (MeOD-*d*_4_/D_2_O) and a maximal shift of 0.84 ppm assigned to peak c’ were identified, indicating the aggregation process was occurring. However, with the continuous increase of D_2_O content, there were solids precipitated out of the solution. Up to 1:4 ratio, little **C9** existed in the solution and the residual signals moved back to the low field being similar to the pattern of starting state in MeOD-*d*_4_. These suggested that **C9** failed to form a stable aggregate in an aqueous solution due to its less sufficient hydrophilicity.

To further confirm the aggregation-induced nanoparticles formed in water, we carried out atomic force microscope (AFM) measurement of **C10**-**C12** in wet conditions^35^ (Fig. 2c and Supplementary Figs. 29–31). Typically, a drop of aqueous solution of **C11** (1.5 mM) was cast on a freshly cleaved mica surface and subjected to test. The pristine AFM images showed uniform spherical nanoparticles dispersed in water. 2D and 3D height profiles gave a particle size around 8 nm. Statistical analysis provided sectional size distribution of **C11**-based nanoparticles with an average diameter of 8.32 nm (Fig. 2d), which is comparable to the DOSY radius (3.98 nm, Supplementary Fig. 5).

At this stage, we could confirm that rather than an expected molecular-level solvated state in solution, these optimized **C10–C12** molecules undergo aggregation and self-assembly process to form nanoparticles in aqueous solution. Thus, the above obtained HSPs for **C10–C12** could not represent the real conditions for these nanoparticles. Meanwhile, it is unworkable to directly obtain their HSPs neither from conventional experimental method because these nanoparticles only exist in aqueous media but disassemble once dissolve in organic solvents, nor from calculations because of the complex and unknown precise nanostructures. However, the compatibility between molecular **C10–C12** and H_2_O disclosed by HSP analysis is a prerequisite to bringing these molecules into water phase as the first step, thus forming the thermodynamic-favored nanoparticles. This phenomenon also well explains why the **C10–C12** holding not small enough but near to boundary condition REDs (0.9–1.0) could show good to excellent aqueous solubility performance.

To gain more insight into the structure and stacking configuration of these nanoparticles, two-dimensional NMR spectroscopy was adopted to in situ investigate this problem. Among versatile NMR technologies, linear nuclear overhauser effect (NOE) growth versus mixing time in a suitable region is used for quantitative distance determination in solution^36^ and expressed by the following equation:

2 $${r}_{{{\rm {AB}}}}={r}_{{{\rm {ref}}}}{({\sigma }_{{{\rm {ref}}}}/{\sigma }_{{{\rm {AB}}}})}^{1/6}$$

where *r*_ref_ is the known interproton distance as reference, *σ*_ref_ and *σ*_AB_ are the NOE growth rates for reference and interprotons (A and B), *r*_AB_ is the unknown distance to be determined.

A series of remote NOE correlations with proton d were specifically observed for **C11** in D_2_O compared with MeOD-*d*_4_ condition, indicative of close packing induced spatial proximity in the aggregation state (Supplementary Fig. 11). The intramolecular protons g and d were selected as the reference and the distance was measured to be 4.08 Å from single crystal analysis. Quantification of the NOE growth rates and introducing to Eq. (2) yield a series of distances between proton d and adjacent phenanthroline protons (Fig. 2e). Together with all the calculated values, we can deduce that the distance between stacked molecules is <5 Å within the nanoparticle structure in water. Considering the height of nanoparticle is about 8.0 nm, there are at least 16 layers of molecular panel stacking among the nanostructure. Identically, **C10** and **C12** share a similar packing mode in an aqueous solution (Supplementary Figs. 15 and 16). Such a large ensemble with a considerable number of planar molecules as subcomponents in water is reminiscent of protein behaviors in solution, which hide their hydrophobic area in the core through winding and folding and expose the hydrophilic surface as exterior to interact with water molecule^8^. Under the same principle, these nanoparticles could reduce the area of hydrophobic parts exposed to water via close packing and forming efficient hydrogen bonding interactions in the interface as proved by the following crystal structure analysis.

### Physicochemical properties of self-assembled nanoparticles

**Fig. 3:**
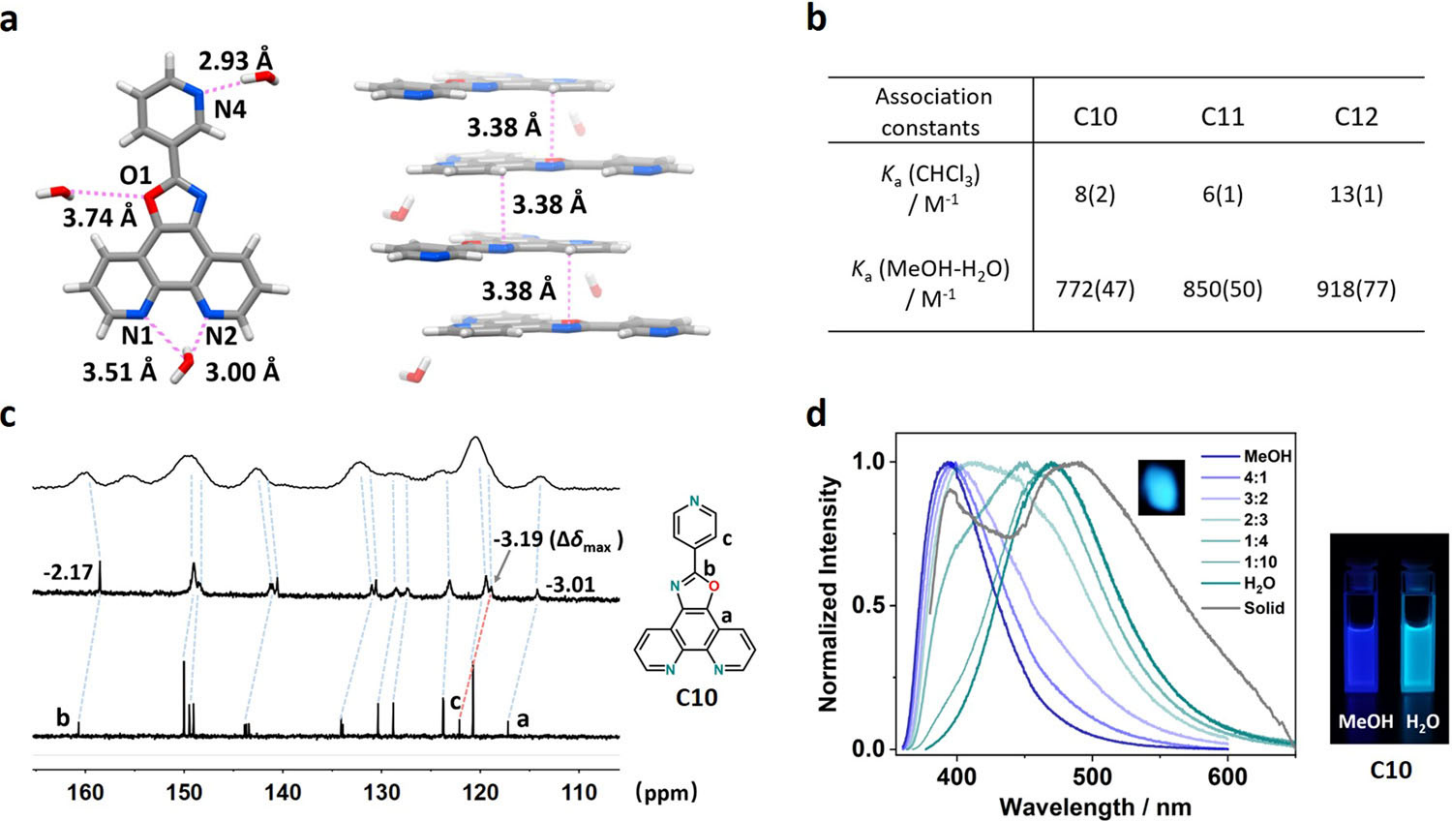
Physiochemical properties of optimized N/O doping molecules with excellent aqueous solubility. **a** Crystal structure of **C11** (left: hydrogen bonding connecting mode around one **C11** molecule, the neighbored N-O distances forming hydrogen bonding were labeled, right: molecular packing along *c*-axis, the interlayers distances were labeled). **b** Apparent association constants for **C10**-**C12** in CHCl_3_ and H_2_O were estimated via ^1^H NMR fitting, respectively. **c** Comparison of ^13^C NMR spectra for **C10** in different existing states (bottom: MeOD-*d*_4_, middle: D_2_O, up: solid-state ^13^C MAS). **d** Left: luminescent emission spectra of **C10** upon 365 nm excitation in solution with viable solvent ratios and solid states (inserted is the photograph of the solid sample when excitation), right: photograph of **C10** emission upon 365 nm excitation in MeOH and H_2_O, respectively.

Attempts to obtain the single crystals from water that are suitable for X-ray diffraction analysis only succeeded for **C11** through slow evaporation (Supplementary Data2). For **C10** and **C12**, the excellent aqueous solubility may be detrimental to crystal growth. Alternatively, both crystals were obtained under evaporation conditions from methanol and chloroform, respectively (Supplementary Data 1, Data 3, Supplementary Table 2). Taking **C11** for instance, displays ideal aromatic planarity from crystal analysis. Remarkably, the hydrogen bonds are ubiquitously existing across the structure. In detail, one **C11** molecule closely contacts three H_2_O via hydrogen bonding interactions (Fig. 3a). Specifically, N2 and N4 atoms as acceptors form relatively strong hydrogen bonds with neighbored water molecules with distances of 3.00 and 2.93 Å and angles of 147.70° and 170.04°, respectively. Relatively weak hydrogen bonds are formed for N1 and O1 with distances of 3.51 and 3.74 Å and angles of 141.13° and 82.48°. In the crystal lattice, the molecular panels adopt a face-to-face packing mode via π-π stacking along the *c*-axis (Fig. 3a). The adjacent layer distance is measured to be 3.38 Å that is shorter than aqueous aggregation conditions, indicative of a more compact packing manner in the crystalline state. The water molecules show a zigzag distribution along the interlayers to act as hydrogen bonding donors. Similar π–π stacking phenomena are also found for **C10** and **C12** in their lattices (Supplementary Fig. 32). These widely existing hydrogen bonds in crystalline states provide direct evidence to visualize the intermolecular interactions and emphasize their significance toward hydrophilicity and aqueous solubility.

As is known, high concentration as a driving force to promote the molecular aggregation in solution is ubiquitous and well recognized^37,38^. To compare the high concentration induced aggregation phenomenon with our case, we measured their apparent association constants (*K*_a_) by ^1^H NMR shift analysis after simplifying the aggregates to a dimeric model^39^ (Supplementary Figs. 17–28). As listed in Fig. 3b, **C10**-**C12** in chloroform display relatively weak association ability induced by high concentration factor. In contrast, their apparent association constants are increased by two orders of magnitude in hydrophilic media, indicative of a more stable and less dissociative ensemble. Meanwhile, the ^13^C NMR spectra gave more informative details about **C10**-**C12** in different existing states (Fig. 3c, Supplementary Figs. 13 and 14). For instance, **C10** displayed characteristic sharp ^13^C nuclear resonance signals in MeOD-*d*_4_. In comparison, these signals became broadened and moved to upfield with a maximal 3.19 ppm shift in D_2_O conditions, which was almost in consistent with the solid-state ^13^C magic-angle spinning (MAS) NMR spectrum despite its much broader signature. Moreover, the luminescent emissions of **C10**-**C12** in various solvent ratios and solid-state were also screened (Fig. 3d, Supplementary Figs. 33 and 34). Along with the water content increasing in MeOH/H_2_O mixture, the emission peak showed a redshift from the ultraviolet region to the blue-green region (e.g., from 393 to 472 nm for **C10**), which may be caused by competitive nonradiative relaxation process between neighbored molecules in close packing fashion. Unexpectedly, the maximal emissions in water are close to the solid state of **C10**-**C12** (e.g., 472 nm in water versus 486 nm in solid for **C10**), indicative of a homologous excited-state energy transfer pathway. We shall speculate that these self-assembled nanoparticles have quasi-solid behaviors in water and possess preliminary solid-like properties from NMR and luminescence perspectives.

## Conclusions

Inspired by the simple aromatic heterocycles with desired hydrophilicity, we have expanded this trend and developed an efficient strategy of precise doping N/O heteroatoms on a predesigned polycyclic aromatic skeleton to greatly enhance its hydrophilicity and aqueous solubility. A series of analogous N/O doping molecules demonstrate that both properties are closely related to the doping species, numbers, and positions. The HSP calculations depending on two sets of water HSP under dilute and dense solutes conditions can give a considerable prediction of improved hydrophilicity and aqueous solubility along with the stepwise N/O doping, which is consistent with the experimental results on the whole. Specifically, the enhanced *δ*_H_ representing hydrogen bonding factor plays a significant role in improving hydrophilic performance. Unexpectedly, three molecules with optimal N/O doping achieve excellent aqueous solubility via a self-assembly process to form nanoparticles. The single crystal X-ray analysis proves the widely existing hydrogen bonding between N/O heteroatoms and water that contribute much to the aqueous solubility performance. Interestingly, these nanoparticles demonstrate quasi-solid properties in water from NMR and luminescence perspectives, which may derive from compact packing and large size of nanoparticles. In brief, we provide a useful mind to construct hydrophilic and even water-soluble polycyclic aromatics and give insight into the mechanism of their self-assembly behaviors in solution.

## Methods

All details on syntheses, solubility-related parameters determination, NMR studies, AFM measurements, single crystal X-ray analysis, and luminescent emission are provided in the Supplementary Information.

## Supplementary information

Li_PR File

Supplementary Information

Description of Additional Supplementary Files

Supplementary data 1

Supplementary data 2

Supplementary data 3

## Data Availability

The data supporting the findings of this study are available within this article and its supplementary information and are also from the corresponding authors upon reasonable request. Crystallographic data for **C10**, **C11** and **C12** are deposited in Supplementary Data1-Data3 files and free of charge from the Cambridge Crystallographic Data Centre (www.ccdc.cam.ac.uk) under reference nos. 2072956, 2072957 and 2072958, respectively.
