# Supplementary material for: Precise heteroatom doping determines aqueous solubility and self-assembly behaviors for polycyclic aromatic skeletons
Source: Commun Chem. 2022 Aug 29;5:104. doi: 10.1038/s42004-022-00724-1 (PMC9814590; doi:10.1038/s42004-022-00724-1)
Supplement: Supplementary file 2 — Supplementary Information [file 42004_2022_724_MOESM2_ESM.pdf]

Supplementary Information

Precise heteroatom doping determines aqueous solubility and self-assembly behaviors for polycyclic aromatic skeleton

Kang Li,[1,*] Jia-Min Hu,[1] Wei-Min Qin,[1] Jing Guo[2] and Yue-Peng Cai[1,*]

[1] School of Chemistry, South China Normal University, Guangzhou 510006, China

[2] School of Chemistry, Sun Yat-Sen University, Guangzhou 510275, China

*e-mail: likang5@mail2.sysu.edu.cn, caiyp@scnu.edu.cn

Contents:

1. Supplementary Methods

   1.1 Materials and methods ……………………………………………………………S2

   1.2 Synthesis and characterization of N/O doped molecules…………………………S3

   1.3 Determination of solubility-related parameters…………………………….… ..S10

2. Supplementary Notes

   2.1 Detailed NMR studies………………………………………………………...S14

   2.2 AFM measurements……………………………………………………….....S39

   2.3 Single crystal X-ray analysis…………………………………………………S42

   2.4 Luminescent emission ……………………………………………………...S45

3. Supplementary References…………………………………………….……….…S47

1. Supplementary Methods

1.1 Materials and methods

Unless otherwise noted, all starting chemicals were used as commercially purchased without further purification. Nuclear magnetic resonance spectra (^{1}H NMR, COSY, NOESY, DOSY, ^{13}C NMR) of liquid samples were recorded on a Bruker AVANCE III 400 (400 MHz for ^{1}H and 100 MHz for ^{13}C) spectrometer and ^{1}H chemical shifts were quoted in parts per million (ppm) relative to the signals corresponding to the residual non-deuterated solvents or 0.0 ppm for tetramethyl silane (TMS). The ^{13}C magic-angle spinning solid-state nuclear magnetic resonance (^{13}C MAS NMR) spectra were recorded on a Bruker AVANCE NEO 600 (150 MHz for ^{13}C nuclei) spectrometer with 4 mm RevNMR-style zirconia rotor. HRESI-TOF mass spectra were measured on Bruker maXis 4G and the data analyses were processed on Bruker Data Analysis software. Single crystal X-ray diffraction data were collected on an Agilent SuperNova X-ray diffractometer using micro-focus X-ray sources (Cu-K_{α}, λ = 1.54184 Å). The water contact angles were measured on the contact angle system OCA 20 (Dataphysics, Germany). The steady-state emission spectra and decay lifetimes were measured on an Edinburgh FLS980 fluorescence spectrophotometer. AFM measurements were carried out on Dimension FastScan Bio atomic force microscope using mica plate as substrate.

1.2 Synthesis and characterization of N/O doped molecules

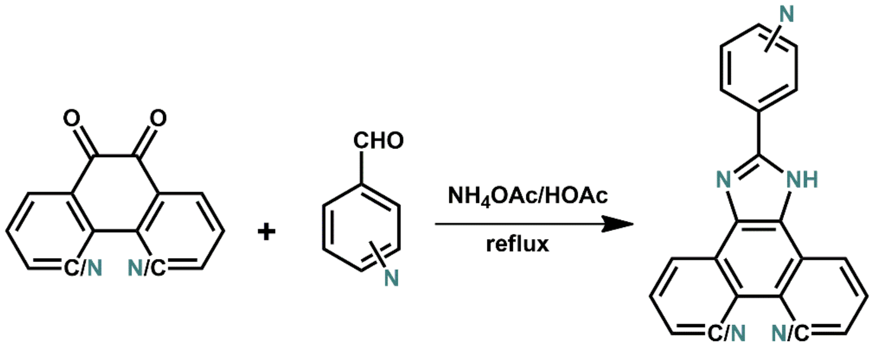

Supplementary Scheme 1. Synthetic route for **C2** to **C6**[1]

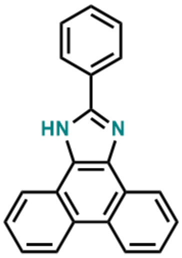

2-phenyl-1*H*-phenanthro[9,10-*d*] imidazole (C2)

To 20 mL of glacial acetic acid were stepwise added phenanthrene-9,10-dione (1.0 g, 4.8 mmol), ammonium acetate (7.2 g, 93.4 mmol) and benzaldehyde (0.6 g, 5.6 mmol). The mixture was refluxed at 120 °C for 3 h. After completion of the reaction, the solution was cooled to room temperature and diluted with water (60 mL). Then, the solution was carefully neutralized with drops of ammonia (25 %) under water bath until large amount of yellowish solid appeared. After filtration, the solid was stepwise washed by water and ethanol, and dried in vacuum to obtain the desired product (1.3 g, 93 % yield). ^{1}H NMR (400 MHz, DMSO-d_6, 25 °C, ppm) δ 13.46 (br, 1H), 8.91 - 8.81 (dd, J_1 = 14.0 Hz, J_2 = 8.3 Hz, 2H), 8.62 - 8.53 (dd, J_1 = 12.8 Hz, J_2 = 8.0 Hz, 2H), 8.34 – 8.29 (d, J = 7.6 Hz, 2H), 7.79 – 7.69 (m, 2H), 7.68 – 7.57 (m, 4H), 7.53 – 7.47 (t, J = 7.2 Hz, 1H). ^{13}C NMR (101 MHz, DMSO-d_6, 25 °C, ppm), δ 149.54, 137.44, 130.83, 129.70, 129.41, 128.15, 128.01, 127.64, 127.55, 127.45, 126.62, 125.84, 125.64, 124.58, 124.22, 122.89, 122.47, 122.35. HRESI-MS Calcd. for $C_{21}H_{15}N_2$ $[M+H]^+$: 295.1235. Found: 295. 1230.

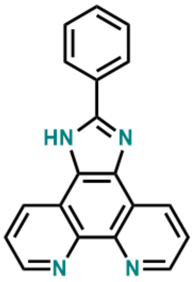

2-phenyl-1*H*-imidazo[4,5-*f*] [1,10] phenanthroline (C3)

The procedure was similar to the above described for compound C2 except phenanthrene-9,10-dione was replaced by 1,10-phenanthroline-5,6-dione as starting material. Yield: 85%. ^{1}H NMR (400 MHz, DMSO-d_6, 25 °C, ppm) δ 13.77 (br, 1H), 9.08 – 9.02 (dd, J_1 = 4.4 Hz, J_2 = 1.6 Hz, 2H), 8.97 - 8.92 (dd, J_1 = 8.0 Hz, J_2 = 1.6 Hz, 2H), 8.33 – 8.27 (d, J = 7.6 Hz, 2H), 7.90 – 7.79 (m, 2H), 7.67 – 7.59 (t, J = 8.0 Hz, 2H), 7.57 – 7.51 (t, J = 7.6 Hz, 1H). ^{13}C NMR (101 MHz, DMSO-d_6, 25 °C, ppm), δ 151.03, 148.31, 144.07, 130.47, 130.09, 129.52, 126.70, 123.80. HRESI-MS Calcd. for $C_{19}H_{13}N_4$ $[M+H]^+$: 297.1140. Found: 297.1131.

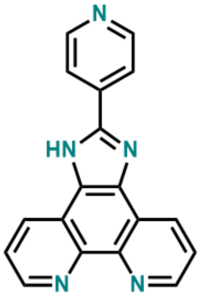

2-(pyridine-4-yl)-1*H*-imidazo[4,5-*f*] [1,10] phenanthroline (C4)

The procedure was similar to the above described for compound C2 except phenanthrene-9,10-dione and benzaldehyde were replaced by 1,10-phenanthroline-5,6-dione and 4-pyridinecarboxaldehyde as starting materials. The crude solid was recrystallized from ethanol to obtain pure product. Yield: 75%.^{1}H NMR (400 MHz, DMSO-d_6, 25 °C, ppm) δ 9.07 – 9.01 (dd, J_1 = 4.4 Hz, J_2 = 1.2 Hz, 2H), 8.94 – 8.88 (dd, J_1 = 8.0 Hz, J_2 = 1.6 Hz, 2H), 8.82 – 8.77 (d, J = 6.0 Hz, 2H), 8.22 – 8.17 (d, J = 6.0 Hz, 2H), 7.87 – 7.80 (dd, J_1 = 8.0 Hz, J_2 = 4.4 Hz, 2H). ^{13}C NMR (101 MHz, DMSO-d_6, 25 °C, ppm), δ 150.94, 148.87, 148.57, 144.29, 137.67, 130.18, 123.87, 122.04, 120.50. HRESI-MS Calcd. for $C_{18}H_{12}N_5$ $[M+H]^+$: 298.1093. Found: 298.1087.

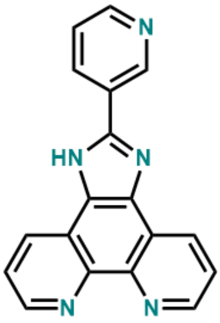

2-(pyridin-3-yl)-1*H*-imidazo[4,5-*f*] [1,10] phenanthroline (C5)

The procedure was similar to the above described for compound C4 except 4-pyridinecarboxaldehyde was replaced by 3-pyridinecarboxaldehyde as starting material. Yield: 80%.). ^{1}H NMR (400 MHz, DMSO-d_6, 25 °C, ppm) δ 14.04 – 13.87 (br, 1H), 9.49 – 9.44 (d, J = 2.0 Hz, 1H), 9.10 – 9.02 (m, 2H), 8.97 – 8.86 (dd, J_1 = 14.8 Hz, J_2 = 8.0 Hz, 2H), 8.74 – 8.69 (d, J = 5.2 Hz, 1H), 8.74 – 8.69 (d, J = 5.2 Hz, 1H), 8.63 – 8.56 (d, J = 8.0 Hz, 1H), 7.92 – 7.79 (m, 2H), 7.70 – 7.63 (dd, J_1 = 8.0 Hz, J_2 = 4.8 Hz, 1H). ^{13}C NMR (101 MHz, DMSO-d_6, 25 °C, ppm), δ 149.58, 148.11, 147.72, 146.77, 143.36, 134.34, 126.42, 124.25, 123.23. HRESI-MS Calcd. for $C_{18}H_{12}N_5$ $[M+H]^+$: 298.1093. Found: 298.1085.

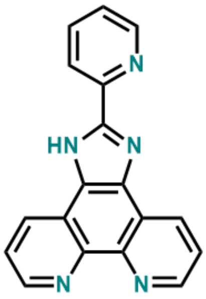

2-(pyridin-2-yl)-1*H*-imidazo[4,5-*f*][1,10]phenanthroline (C6)

The procedure was similar to the above described for compound C4 except 4-pyridinecarboxaldehyde was replaced by 2-pyridinecarboxaldehyde as starting material. Yield: 50%. ^{1}H NMR (400 MHz, $CDCl_3$, 25 °C, ppm) δ 9.27 – 9.23 (d, J = 4.0 Hz, 2H), 9.12 – 9.05 (dd, J_1= 8.0 Hz, J_2= 1.8 Hz, 1H), 8.93 – 8.91 (d, J = 4.8 Hz, 1H), 8.90 – 8.87 (dd, J_1 = 8.0 Hz, J_2 = 1.8 Hz, 1H), 8.47 – 8.39 (d, J = 8.0 Hz, 1H), 7.99 – 7.95 (d, J = 8.0 Hz, 1H), 7.80 – 7.75 (m, 2H), 7.55 – 7.48 (dd, J_1= 8.0 Hz, J_2= 4.8 Hz, 1H). ^{13}C NMR (101 MHz, $CDCl_3$, 25 °C, ppm), δ 162.23, 150.67, 150.33, 145.04, 145.90, 145.32, 145.01, 144.12, 137.58, 134.90, 130.98, 129.25, 125.78, 123.89, 123.51, 123.62, 123.05, 118.11. HRESI-MS Calcd. for $C_{18}H_{12}N_5$ $[M+H]^+$: 298.1093. Found: 298.1089.

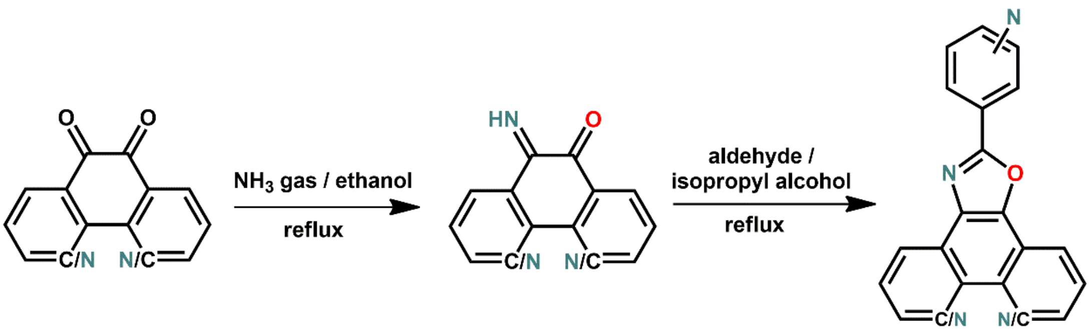

Supplementary Scheme S2. Synthetic route for **C7** to **C12**[2]

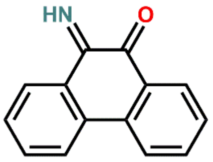

10-iminophenanthren-9(*10H*)-one (P1)

To 20 mL of ethanol and 5 mL of chloroform were added phenanthrene-9,10-dione (1.0 g, 4.8 mmol) and the mixture was bubbled with ammonia gas at 90°C for 0.5 h. The solution was concentrated under reduced pressure to obtain the yellow solid as desired product (0.98 g, 98 % yield). ^{1}H NMR (400 MHz, $CDCl_3$, 25°C, ppm) δ 11.69 (s, 1H), 8.54 (d, J = 4.0 Hz, 1H), 8.29 (d, J = 8.0 Hz, 1H), 8.14 - 8.04 (dd, J_1 = 16.0 Hz, J_2 = 8.0 Hz, 2H), 7.79 - 7.71 (t, J = 8.0 Hz, 1H), 7.67 – 7.60 (t, J = 8.0 Hz, 1H), 7.53 – 7.44 (dd, J_1 = 16.0 Hz, J_2 = 8.0 Hz, 2H). ^{13}C NMR (101 MHz, $CDCl_3$, 25 °C, ppm), δ 183.6, 152.1, 132.0, 131.5, 130.6, 130.0, 129.4, 128.5, 127.3, 125.8, 122.3. HRESI-MS Calcd. for $C_{14}H_{10}NO$ $[M+H]^+$: 208.0762. Found: 208.0771.

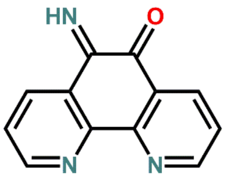

6-imino-1,10-phenanthrolin-5(*6H*)-one (P2)

The procedure was similar to the above described for compound P1 except phenanthrene-9,10-dione was replaced by 1,10-phenanthroline-5,6-dione as starting material. Yield: 97 %. ^{1}H NMR (400 MHz, $CDCl_3$, 25 °C, ppm) δ 11.98 (s, 1H), 9.19 – 9.12 (dd, J_1 = 4.0 Hz, J_2 = 2.0 Hz, 1H), 9.09 – 9.03 (dd, J_1 = 8.0 Hz, J_2 = 2.0 Hz, 1H), 8.87 – 8.81 (dd, J_1 = 8.0 Hz, J_2 = 1.6 Hz, 1H), 8.62 – 8.55 (dd, J_1 = 8.0 Hz, J_2 = 1.6 Hz, 1H), 7.61 – 7.53 (m, 2H). ^{13}C NMR (101 MHz, $CDCl_3$, 25 °C, ppm), δ 184.2, 156.1, 155.3, 152.3, 150.6, 139.2, 137.5, 131.8, 125.1, 122.2, 121.4. HRESI-MS Calcd. for $C_{14}H_{10}NO$ $[M+H]^+$: 210.0667. Found: 210.0673.

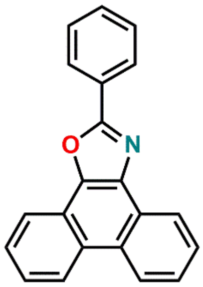

2-phenylphenanthro[9,10-*d*]oxazole (C7)

To 50 mL of isopropyl alcohol were stepwise added 10-iminophenanthren-9(10*H*)-one (1.0 g, 4.8 mmol), benzaldehyde (0.6 g, 5.6 mmol) and piperidine (0.5 mL, 5.0 mmol). The mixture was refluxed at 90 °C for 12 h and evaporated under reduced pressure. The crude product was purified through column silicon gel using $CHCl_3$ as eluent. Yield: 83 %. ^{1}H NMR (400 MHz, $CDCl_3$, 25 °C, ppm) δ 8.82 – 8.74 (t, J = 9.2 Hz, 2H), 8.70 – 8.65 (d, J = 8.0 Hz, 1H), 8.44 – 8.40 (dd, J_1= 8.0 Hz, J_2= 1.6 Hz, 2H), 8.40 – 8.37 (m, 1H), 7.82 – 7.68 (m, 4H), 7.64 – 7.53 (m, 3H).). ^{13}C NMR (101 MHz, $CDCl_3$, 25 °C, ppm), δ 130.93, 129.35, 128.92, 127.62, 127.44, 127.29, 127.19, 126.42, 126.24, 126.16, 123.78, 123.44, 122.97, 121.13, 120.88. HRESI-MS Calcd. for $C_{21}H_{14}NO$ $[M+H]^+$: 296.1075. Found: 298.1081.

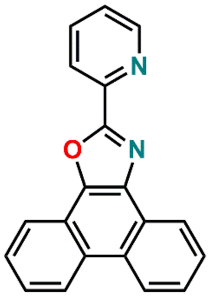

2-(pyridine-2-yl)phenanthro[9,10-*d*]oxazole (C8)

The procedure was similar to the above described for compound C7 except benzaldehyde was replaced by 2-pyridinecarboxaldehyde as starting material. Yield: 65 %. ^{1}H NMR (400 MHz, $CDCl_3$, 25 °C, ppm) δ 9.62 – 9.58 (d, J = 2.4 Hz, 1H), 8.81 – 8.72 (m, 3H), 8.66 – 8.61 (d, J = 8.0 Hz, 2H), 8.39 – 8.34 (dd, J_1= 7.2 Hz, J_2= 2.0 Hz, 1H), 7.81 – 7.68 (m, 4H), 7.54 – 7.48 (dd, J_1= 8.4 Hz, J_2= 4.8 Hz, 1H). ^{13}C NMR (101 MHz, $CDCl_3$, 25 °C, ppm), δ 159.71, 151.45, 148.37, 145.25, 134.23, 129.61, 129.04, 127.61, 127.45, 126.82, 126.44, 126.01, 123.93, 123.84, 123.71, 123.50, 122.96, 120.99, 120.89. HRESI-MS Calcd. for $C_{20}H_{12}N_2ONa$ $[M+Na]^+$: 319.0847. Found: 319.0853.

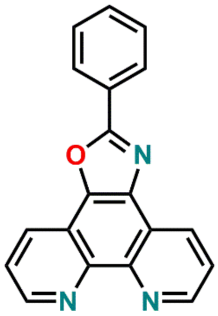

2-phenyloxazolo[4,5-*f*][1,10]phenanthroline (C9)

The procedure was similar to the above described for compound C7 except 10-iminophenanthren-9(10*H*)-one was replaced by 6-imino-1, 10-phenanthrolin-5(6*H*)-one as starting material. Yield: 81 %. ^{1}H NMR (400 MHz, MeOD-d_4, 25 °C, ppm) δ 8.83 – 8.74 (dd, J_1= 10.0 Hz, J_2= 4.4 Hz, 2H), 8.36 – 8.27 (d, J = 8.4 Hz, 1H), 8.17 – 8.09 (d, J = 8.4 Hz, 1H), 7.99 – 7.90 (d, J = 7.2 Hz, 2H), 7.58 – 7.42 (m, 5H). ^{13}C NMR (101 MHz, MeOD-d_4, 25 °C, ppm), δ 163.17, 148.57, 148.40, 143.26, 142.97, 142.22, 133.46, 131.35, 130.03, 128.62, 128.16, 126.68, 125.99, 123.30, 121.90, 117.02. HRESI-MS Calcd. for $C_{19}H_{12}N_3O$ $[M+H]^+$: 298.0980. Found: 298.0985.

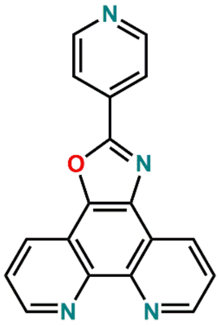

2-(pyridin-4-yl)oxazolo[4,5-*f*][1,10]phenanthroline (C10)

The procedure was similar to the above described for compound C7 except 10-iminophenanthren-9(10*H*)-one and benzaldehyde were replaced by 6-imino-1, 10-phenanthrolin-5(6*H*)-one and 4-pyridinecarboxaldehyde as starting materials. Yield: 76 %. ^{1}H NMR (400 MHz, MeOD-d_4, 25 °C, ppm) δ 8.99 – 8.91 (dd, J_1= 11.6 Hz, J_2= 4.4 Hz, 2H), 8.81 – 8.76 (d, J = 4.4 Hz, 2H), 8.58 – 8.54 (dd, J_1= 8.0 Hz, J_2= 1.6 Hz, 1H), 8.47 – 8.42 (dd, J_1= 8.0 Hz, J_2= 1.6 Hz, 1H), 8.09 – 8.04 (d, J = 4.0 Hz, 2H), 7.74 – 7.65 (m, 2H). ^{13}C NMR (101 MHz, MeOD-d_4, 25 °C, ppm), δ 160.73, 149.95, 149.45, 148.99, 143.85, 143.70, 143.43, 134.08, 133.93, 130.33, 128.82, 123.78, 123.72, 122.11, 120.73, 117.18. HRESI-MS Calcd. for $C_{18}H_{10}N_4ONa$ $[M+Na]^+$: 321.0752. Found: 321.0748.

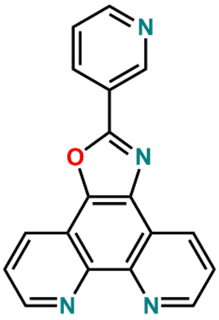

2-(pyridin-3-yl)oxazolo[4,5-*f*][1,10]phenanthroline (C11)

The procedure was similar to the above described for compound C7 except 10-iminophenanthren-9(10*H*)-one and benzaldehyde were replaced by 6-imino-1, 10-phenanthrolin-5(6*H*)-one and 3-pyridinecarboxaldehyde as starting materials. Yield: 82 %. ^{1}H NMR (400 MHz, MeOD-d_4, 25 °C, ppm) δ 9.17 – 9.09 (s, 1H), 8.91 – 8.78 (m, 2H), 8.74 – 8.66 (d, J = 4.4 Hz, 1H), 8.42 – 8.38 (d, J = 8.4 Hz, 1H), 8.37 – 8.32 (d, J = 8.4 Hz, 1H), 8.29 – 8.22 (d, J = 8.0 Hz, 1H), 7.64 – 7.52 (m, 3H). ^{13}C NMR (101 MHz, MeOD-d_4, 25 °C, ppm), δ 162.85, 153.46, 151.32, 150.97, 149.42, 145.71, 145.64, 145.04, 136.89, 135.82, 132.43, 130.75, 126.49, 125.81, 125.33, 124.15, 119.28. HRESI-MS Calcd. for $C_{18}H_{11}N_4O$ $[M+H]^+$: 299.0933. Found: 299.0929.

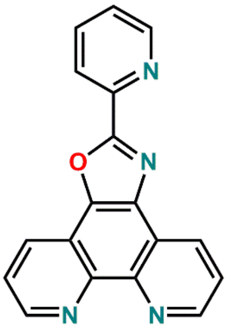

2-(pyridin-2-yl)oxazolo[4,5-*f*][1,10]phenanthroline (C12)

The procedure was similar to the above described for compound C7 except 10-iminophenanthren-9(10*H*)-one and benzaldehyde were replaced by 6-imino-1, 10-phenanthrolin-5(6*H*)-one and 2-pyridinecarboxaldehyde as starting materials. Yield: 78 %. ^{1}H NMR (400 MHz, MeOD-d_4, 25 °C, ppm) δ 8.99 – 8.96 (dd, J_1= 4.4 Hz, J_2= 1.6 Hz, 1H), 8.95 – 8.92 (dd, J_1= 4.4 Hz, J_2= 1.6 Hz, 1H), 8.80 – 8.76 (d, J = 4.4 Hz, 2H), 8.59 – 8.54 (dd, J_1= 8.4 Hz, J_2= 1.6 Hz, 1H), 8.47 – 8.43 (dd, J_1= 8.0 Hz, J_2= 1.6 Hz, 1H), 8.10 – 8.05 (d, J = 4.4 Hz, 2H), 7.75 – 7.66 (m, 2H). ^{13}C NMR (101 MHz, MeOD-d_4, 25 °C, ppm), δ 160.69, 149.99, 149. 45, 149.01, 143.87, 143.71, 143.41, 134.05, 133.92, 130.32, 128.82, 123.77, 123.71, 122.12, 120.70, 117.18. HRESI-MS Calcd. for $C_{18}H_{11}N_4O$ $[M+H]^+$: 299.0933. Found: 299.0927.

1.3 Determination of solubility-related parameters

(1) Measurement of aqueous solubility

We applied the classic shake-flask method to determine the solubility data of **C2–C12** in water. Typically, an excess amount of **C2** was added to 1.0 mL of D_2O, and the mixture was stirred at room temperature for 24 h. After that, the phase separation of saturated solution was finished through centrifugation. ^{1}H NMR technology was applied to quantitatively analysis the concentration of solute using tetrahydrofuran as internal standard. Actually, we didn't synthesize **C1** to obtain its solubility data from experimental method because it's obvious that this compound would be insoluble in water from reasonable judgement. Alternatively, due to its high hydrophobicity and measured water solubility data from **C2–C8** counterpart, we can estimate this value must be at least lower than 0.1 mg / mL as well.

(2) Determination of Hansen solubility parameters (HSP)[3]

A collection of solvents was used to estimate the HSPs values of **C2–C12**, which includes dichloromethane, chloroform, toluene, diethyl ether, hexane, dioxane, pyridine, tetrahydrofuran, dimethyl sulfoxide, N, N-dimethyl formamide, acetone, acetonitrile, methanol and ethanol. If the specific solvent (0.5 mL) can dissolve at least 1.0 mg of certain solute would be rated as good solvent, otherwise as bad solvent. For **C1**, it's expected that this molecule can only dissolve in nonpolar solvents (recrystallized from CCl_4 according to literature report). We inferred that CCl_4, $CHCl_3$ and benzene would be good solvents for **C1**, in contrast, acetonitrile, methanol, ethanol and water would be bad solvents. These rated solvents datasets for **C1–C12** were imported to *HSPiP* software that developed by Dr. Hansen, Abbott and Hiroshi to optimize and obtain three partial HSPs values.

Supplementary Table 1. Collected water contact angles for **C2** – **C12**

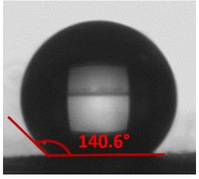	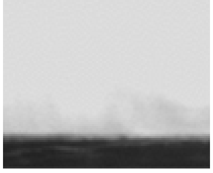	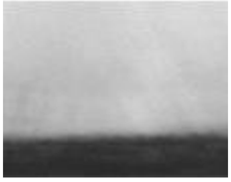	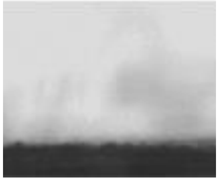
**C2**: 140.6°	**C3**: 0°	**C4**: 0°	**C5**: 0°
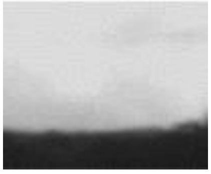	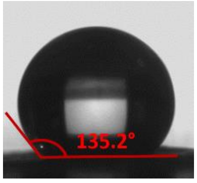	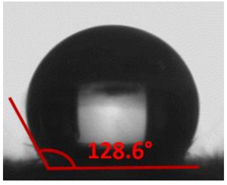	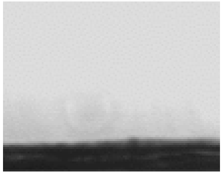
**C6**: 0°	**C7**: 135.2°	**C8**: 128.6°	**C9**: 0°
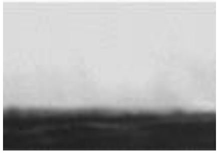	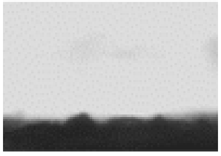	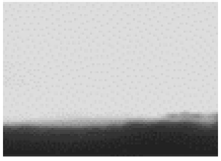	
**C10**: 0°	**C11**: 0°	**C12: 0°**	

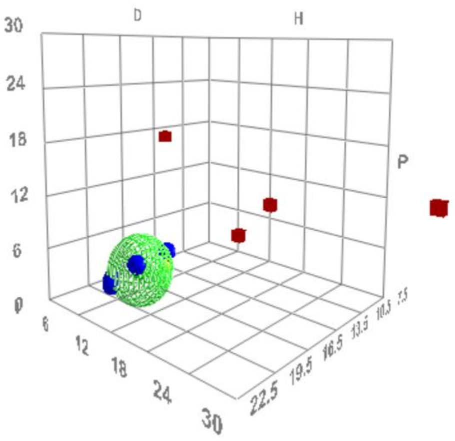

C1

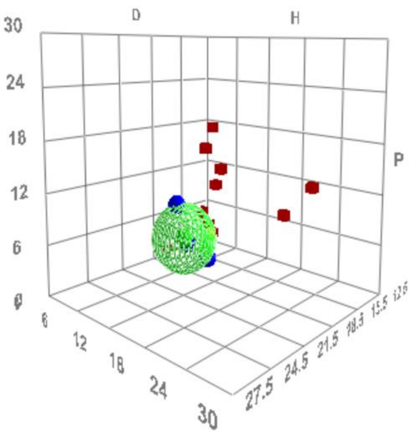

C2

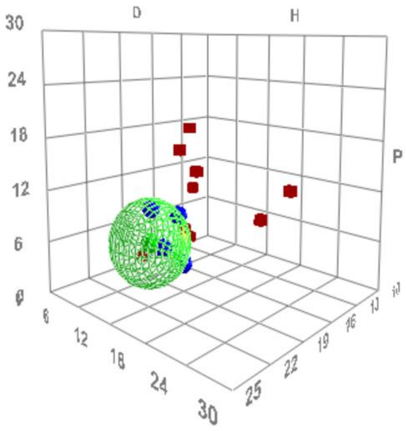

C3

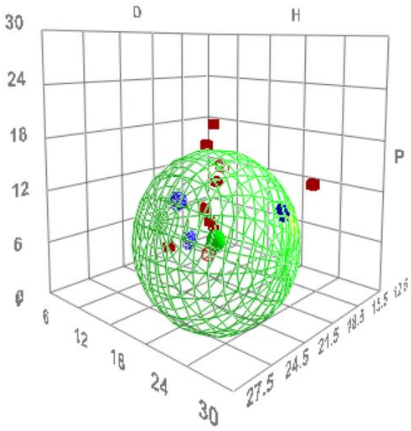

C4

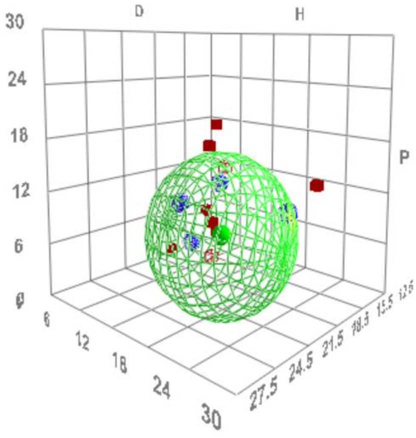

C5

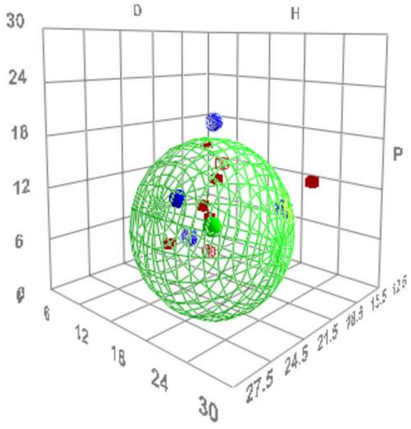

C6

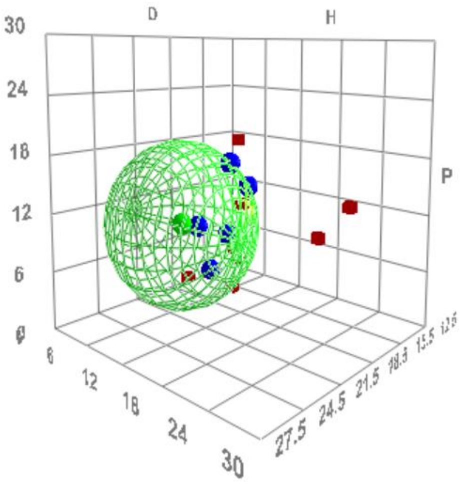

C7

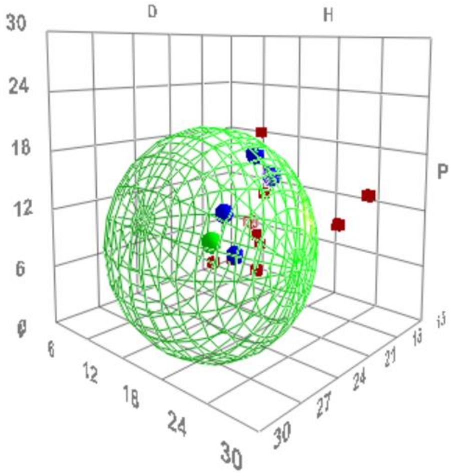

C8

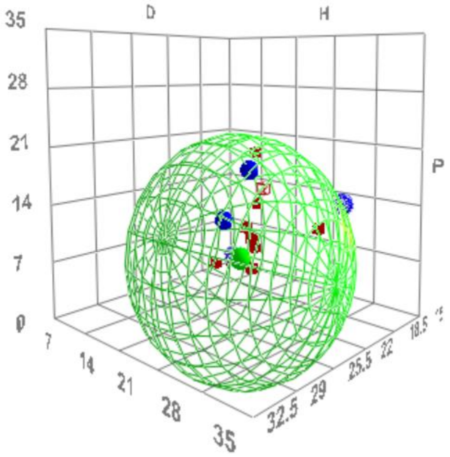

C9

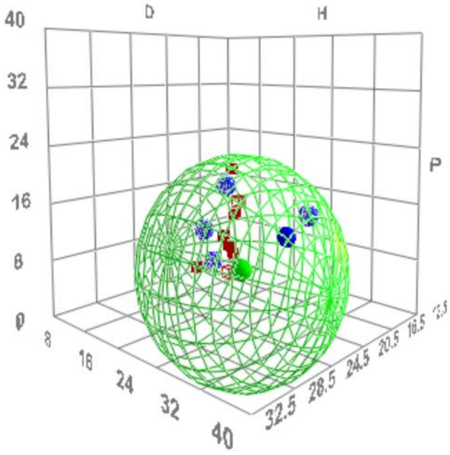

C10

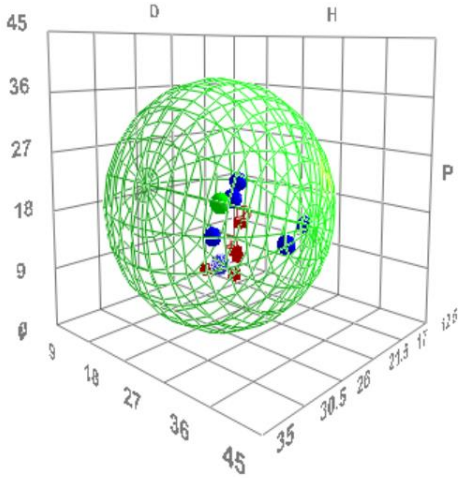

C11

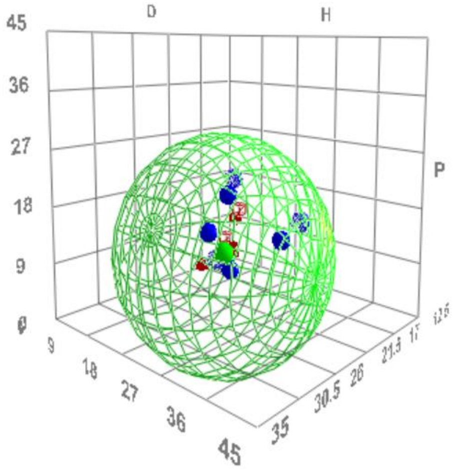

C12

Supplementary Fig. 1. Optimized solubility spheres in Hanse space for **C1-C12** by HSPiP 5.1.03 software (the red cubes represent bad solvents out of the sphere, the blue spheres represent good solvents inside the sphere).

2. Supplementary Notes

2.1 Detailed NMR studies

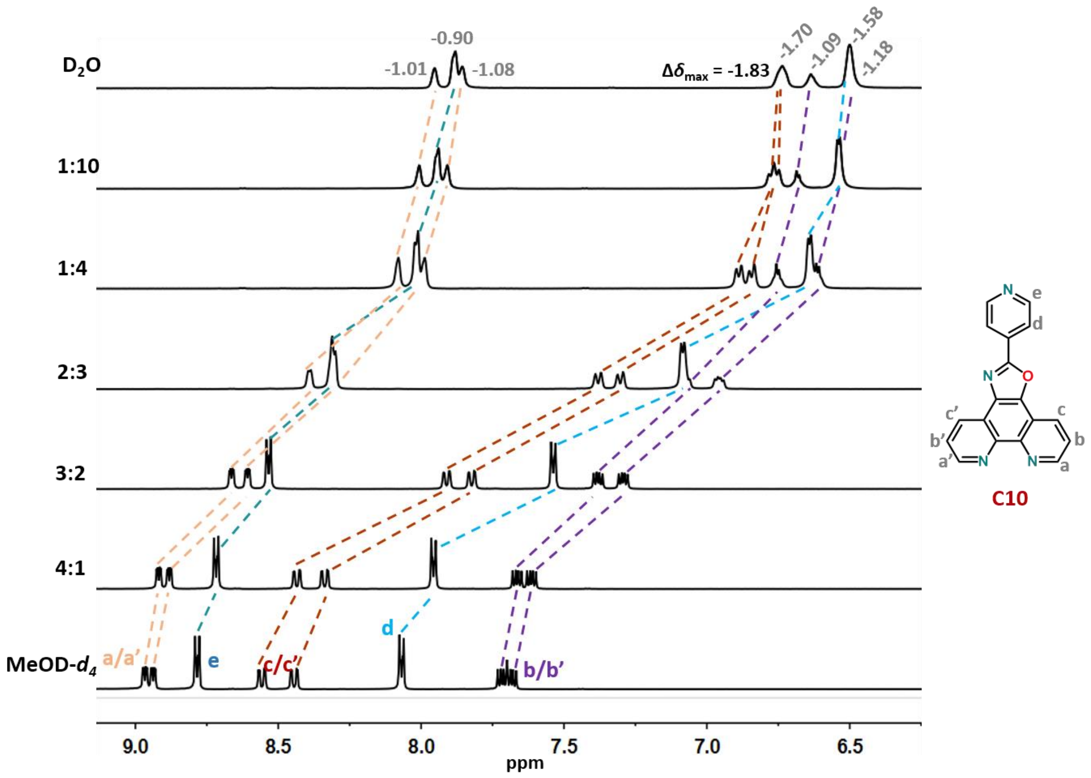

Supplementary Fig. 2. Stacked ^{1}H NMR spectra of **C10** in pure MeOD-d_4 (bottom), D_2O (top) and solvents mixture with distinct volume ratio.

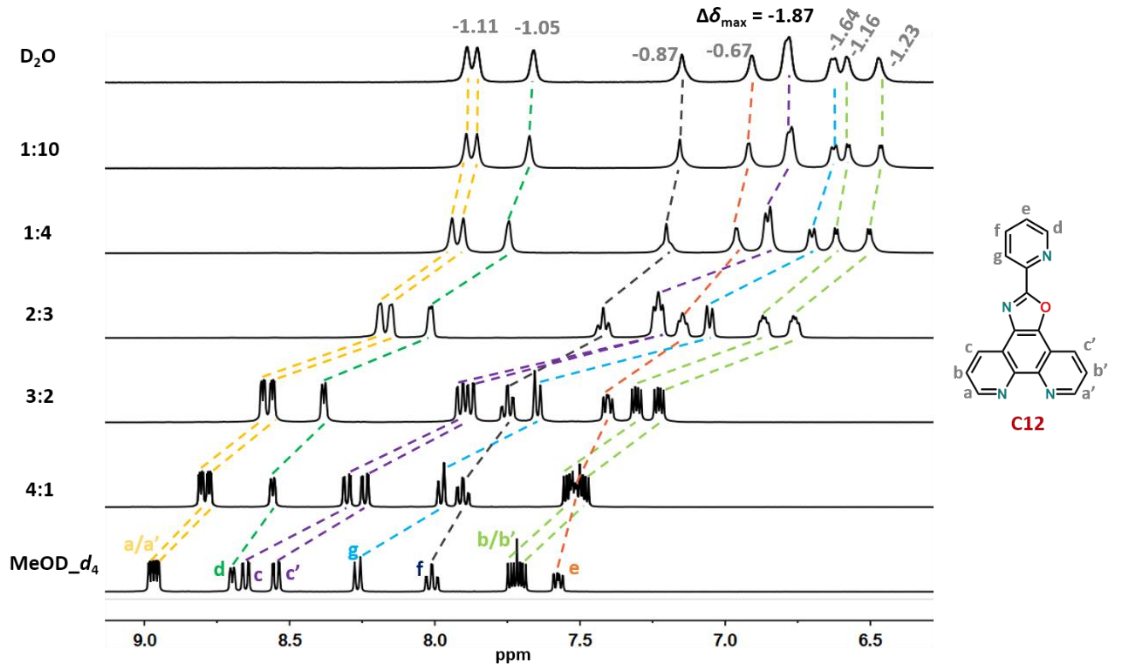

Supplementary Fig. 3. Stacked ^{1}H NMR spectra of **C11** in pure MeOD-d_4 (bottom), D_2O (top) and solvents mixture with distinct volume ratio.

DOSY spectra

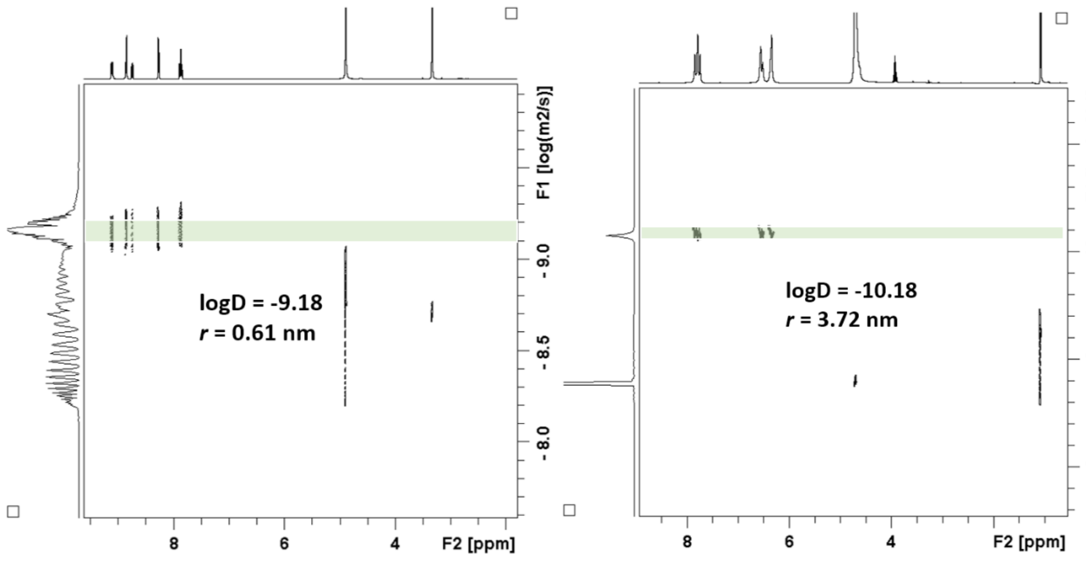

a b

Supplementary Fig. 4. ^{1}H DOSY spectra of **C10** in MeOD-d_4 (a) and D_2O (b) at room temperature.

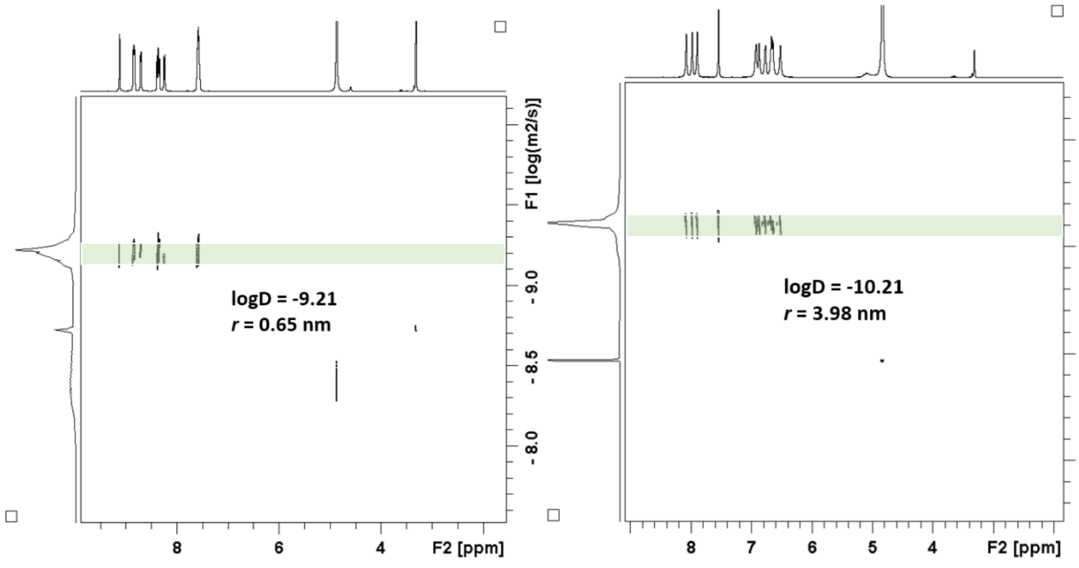

a b

Supplementary Fig. 5. ^{1}H DOSY spectra of **C11** in MeOD-d_4 (a) and D_2O (b) at room temperature.

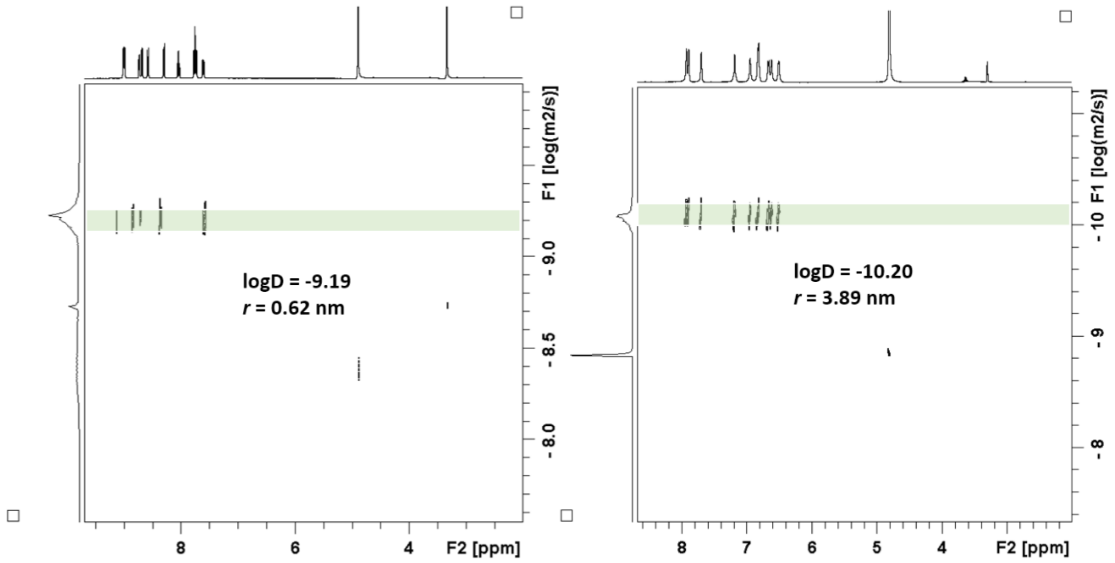

a **b**

Supplementary Fig. 6. ^{1}H DOSY spectra of **C12** in MeOD-d_4 (a) and D_2O (b) at room temperature.

COSY spectra

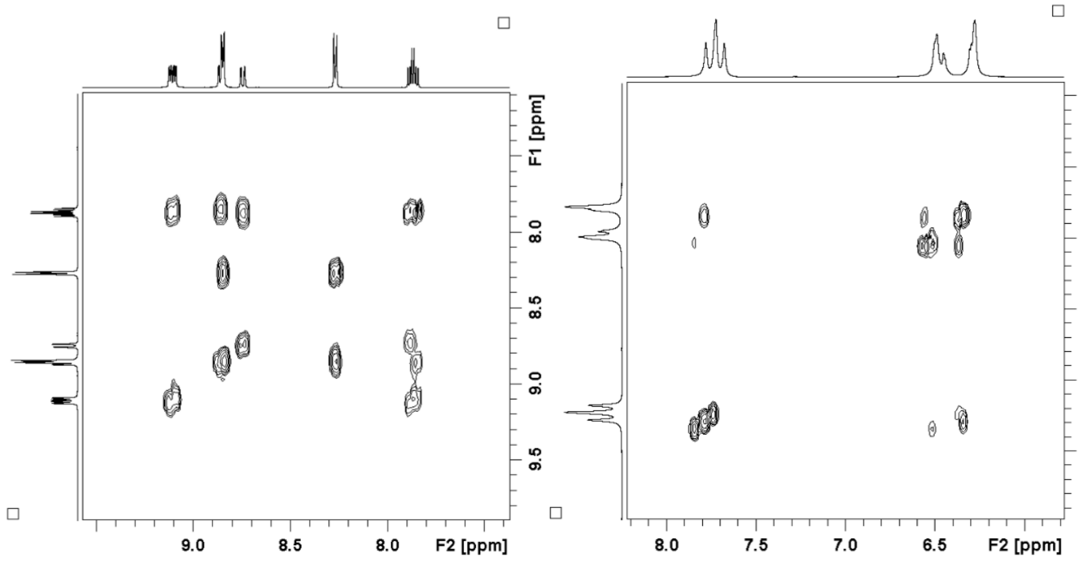

a **b**

Supplementary Fig. 7. ^{1}H-^{1}H COSY spectra of **C10** in MeOD-d_4 (a) and D_2O (b) at room temperature.

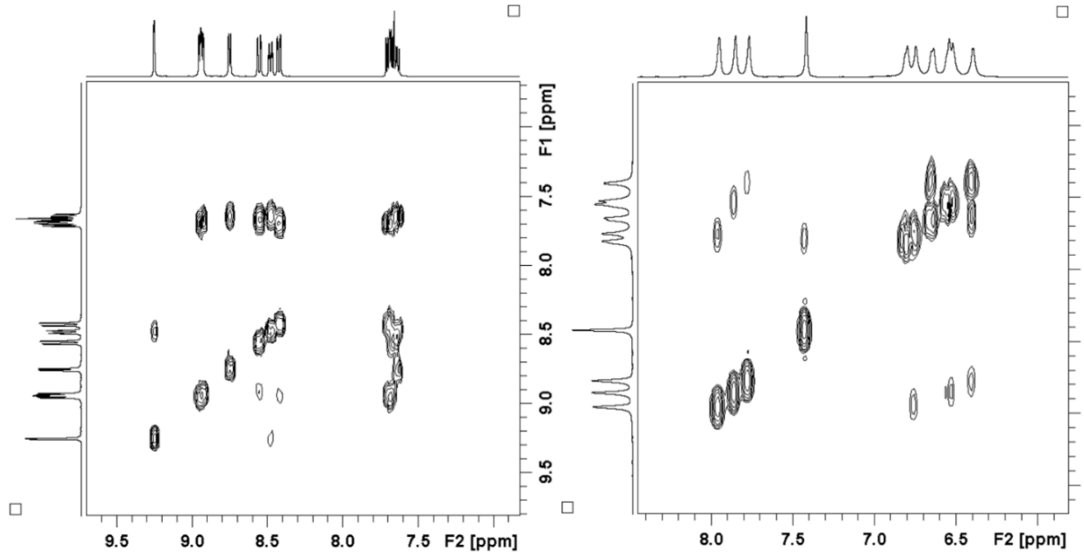

a **b**

Supplementary Fig. 8. ^{1}H-^{1}H COSY spectra of **C11** in MeOD-d_4 (a) and D_2O (b) at room temperature.

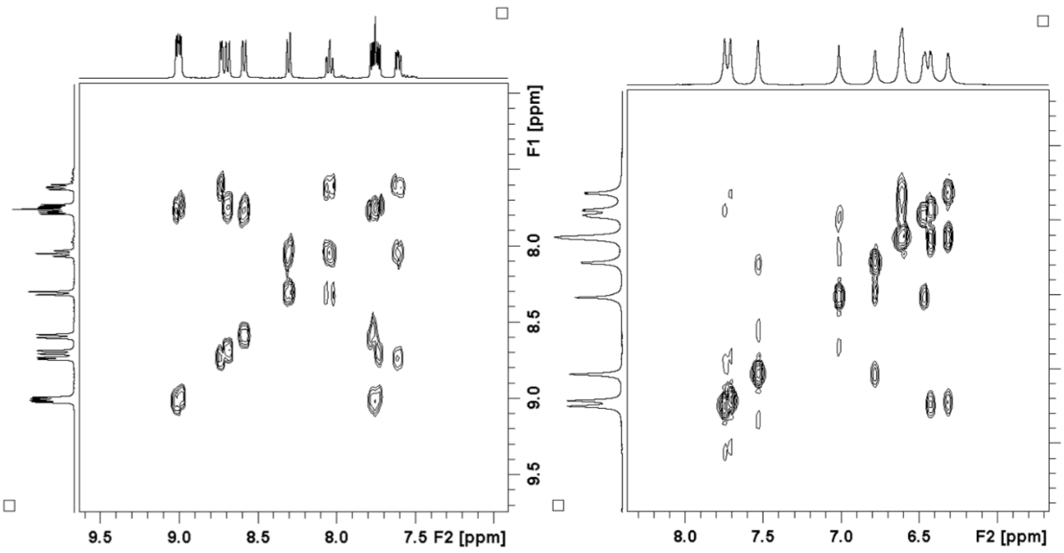

a **b**

Supplementary Fig. 9. ^{1}H-^{1}H COSY spectra of **C12** in MeOD-d_4 (a) and D_2O (b) at room temperature.

NOESY spectra

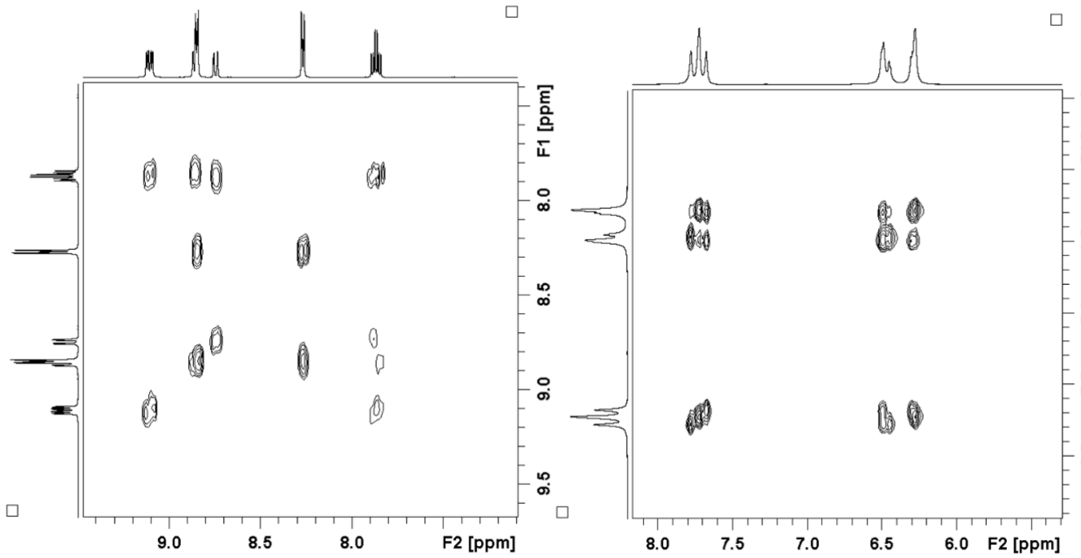

a **b**

Supplementary Fig. 10. ^{1}H-^{1}H NOESY spectra of **C10** in MeOD-d_4 (a) and D_2O (b) at room temperature.

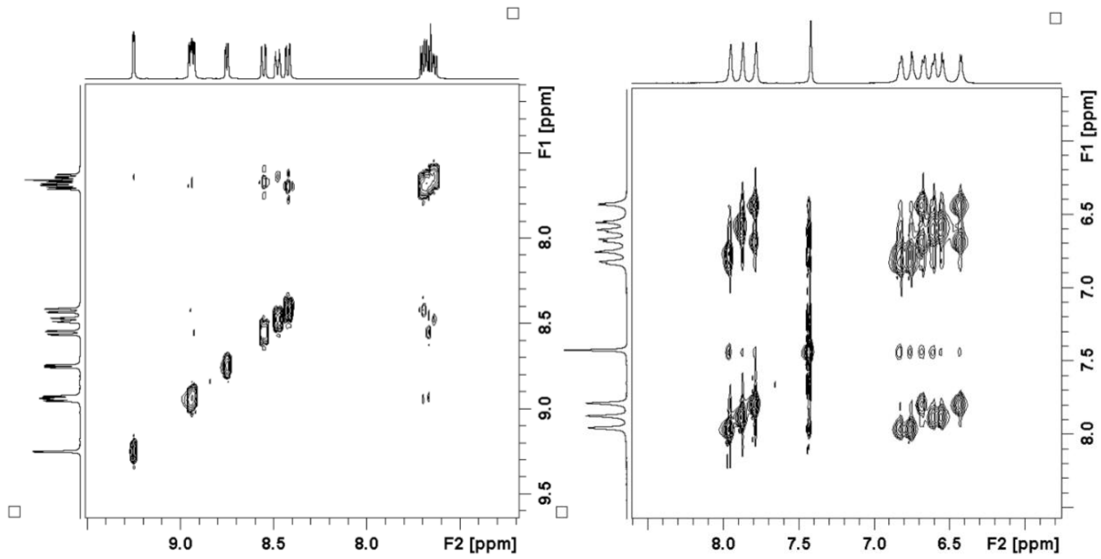

a **b**

Supplementary Fig. 11. ^{1}H-^{1}H NOESY spectra of **C11** in MeOD-d_4 (a) and D_2O (b) at room temperature.

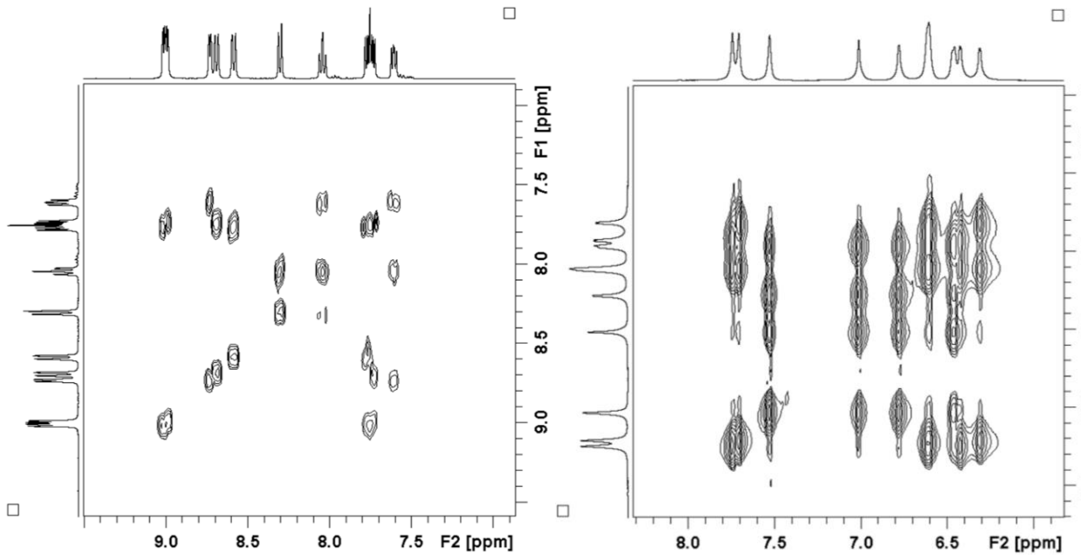

a **b**

Supplementary Fig. 12. ^1H-^1H NOESY spectra of **C12** in MeOD-d_4 (a) and D_2O (b) at room temperature.

^{13}C spectra

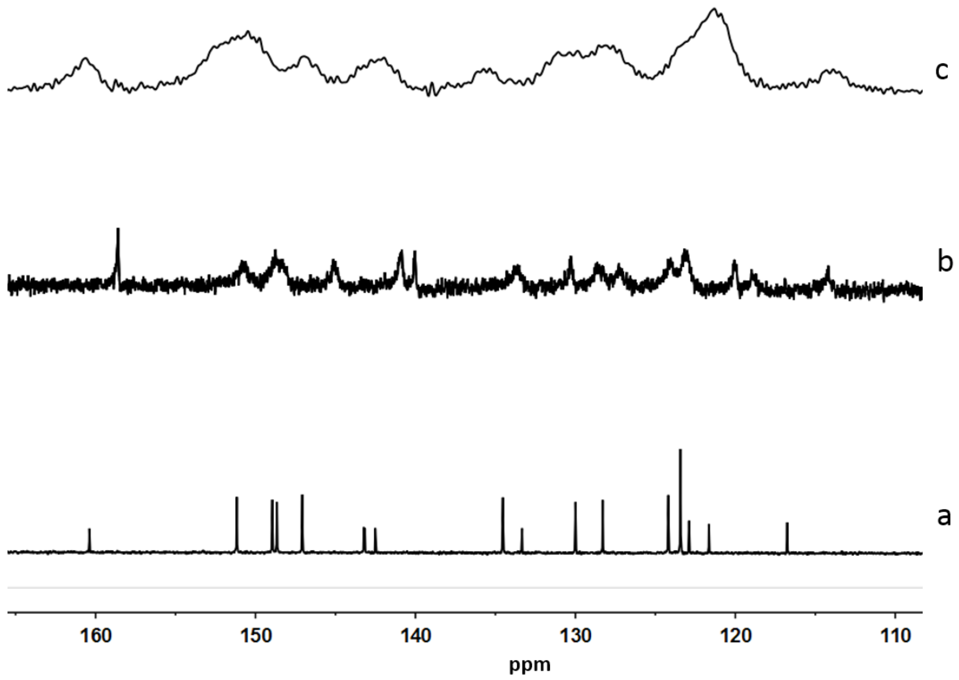

Supplementary Fig. 13. Comparison of ^{13}C NMR spectra for **C11**: (a) MeOD-d_4; (b) D_2O; (c) solid-state ^{13}C MAS.

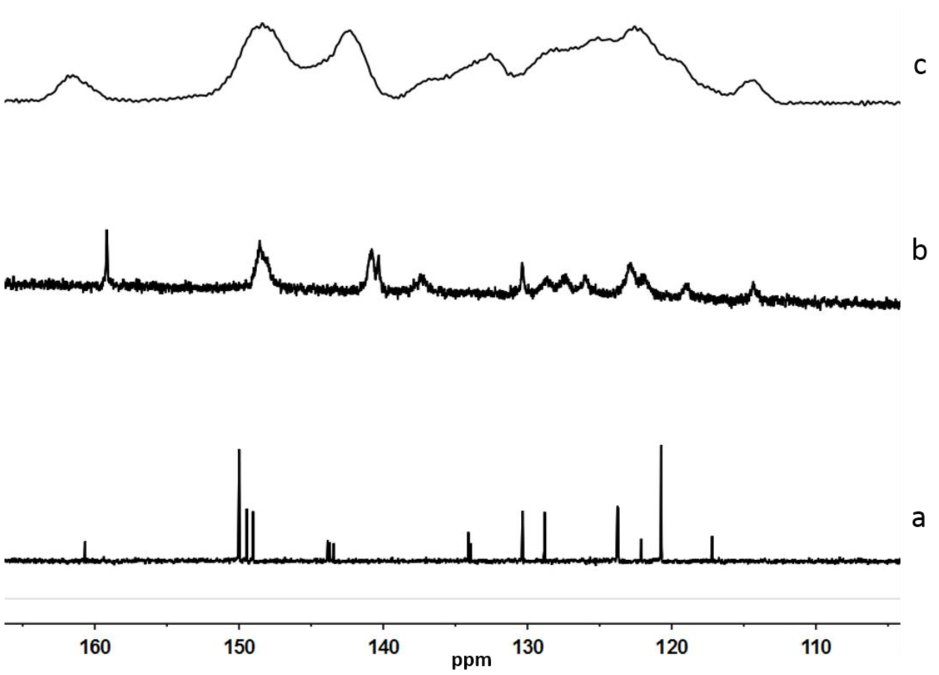

Supplementary Fig. 14. Comparison of ^{13}C NMR spectra for **C12**: (a) MeOD-d_4; (b) D_2O; (c) solid-state ^{13}C MAS.

Quantitative distance determination by NOESY

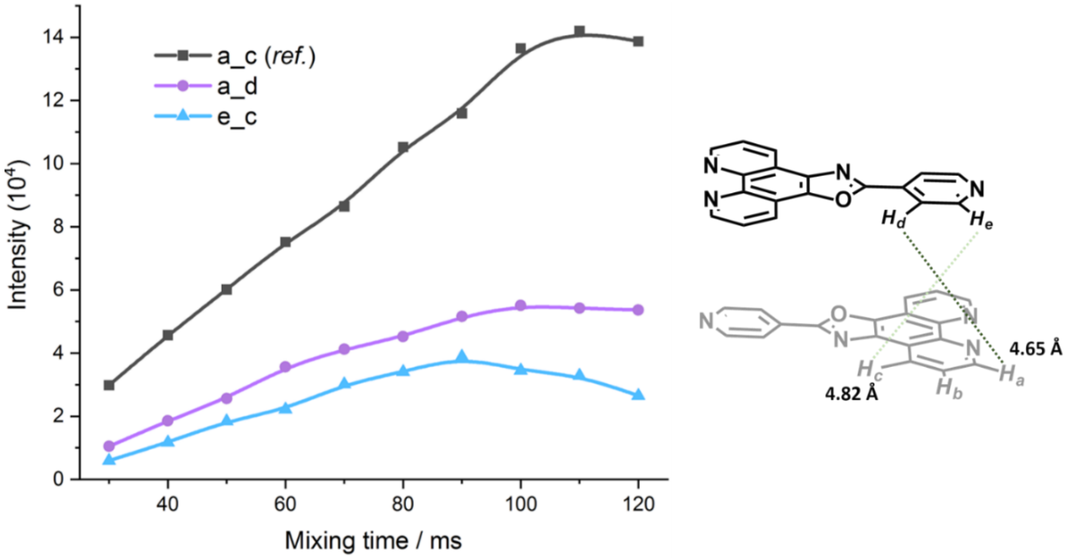

Supplementary Fig. 15. NOE build-up curves for **C10**. Right: the calculated intermolecular proton distances derived from the NOE growth rates analysis using cross-peak of a_c correlation as internal reference.

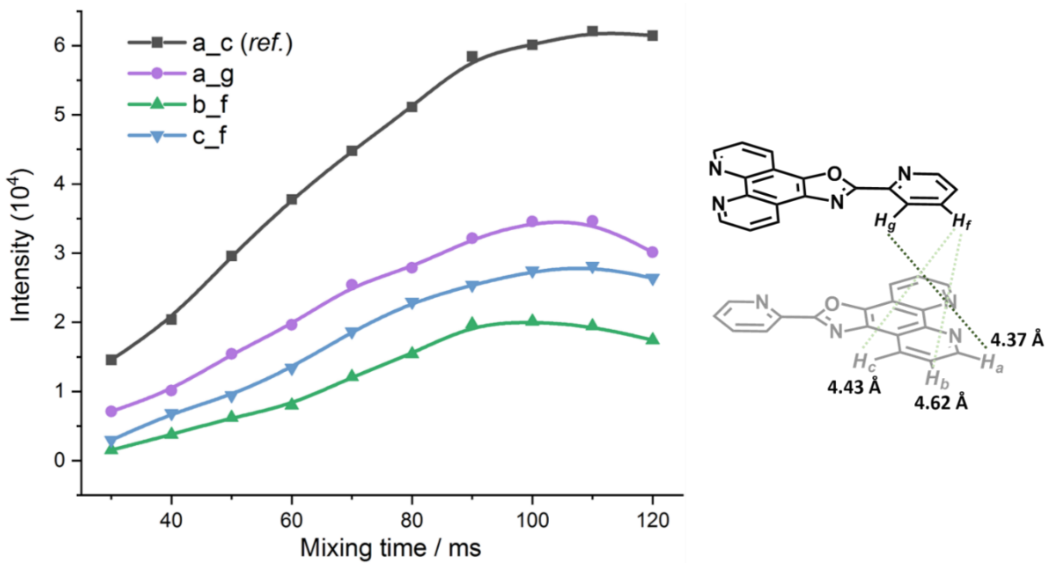

Supplementary Fig. 16. NOE build-up curves for **C12**. Right: the calculated intermolecular proton distances derived from the NOE growth rates analysis using cross-peak of a_c correlation as internal reference.

Association constants (K_a) determination by ^{1}H NMR fitting

The method described below is developed Horman and Dreux.[4] The monomer and dimer exchange is considered as the major equilibrium process for simplification, thus the following is assumed :

$$2M \overset{K_a}{\Leftrightarrow} M_2$$

Where M represents the monomer, M_2 represents the dimer and K_a is the association constant.

So, $K_a = \frac{[M_2]}{[M]^2} = \frac{[M_2]}{(C_0-2[M_2])^2}$ (supplementary eq. 1)

in which, C_0 is the total concentration of the monomer.

After rearrangement, eq. 1 can be rewritten as:

$$\frac{1}{2C_0K_a} = \frac{2[M_2]}{C_0} + \frac{C_0}{2[M_2]} - 2 \qquad \text{(supplementary eq. 2)}$$

If $\frac{2[M_2]}{C_0}$ is denoted as x, $\frac{1}{2C_0K_a}$ is denoted as y, eq. 2 can be transformed to another form

$$y = x + \frac{1}{x} - 2 \qquad \text{(supplementary eq. 3)}$$

Thus, the parameter x represents the fraction of species as dimer. For $0 < x < 1$, eq. 3 can be transformed to

$$x = \left(1+\frac{y}{2}\right) - \sqrt{(1+\frac{y}{2})^2 - 1} \qquad \text{(supplementary eq. 4)}$$

Instead of distinguishable NMR signals for monomer and dimer, in most case, an averaged single set of chemical shift is appeared. Therefore, it is assumed that the measured chemical shift is the weighted average between the monomer and dimer, then

$$\delta_i = \delta_0 - x(\delta_0 - \delta_d) \qquad \text{(supplementary eq. 5)}$$

where δ_i is the measured chemical shift at certain concentration, while δ_0 and δ_d are limiting chemical shifts of monomer and dimer, respectively.

In practical, one should first assume a K_a value as initial, then y is confirmed. Using eq. 4, one can obtain x as the fraction of species as dimer. According to eq. 8, the calculated δ_i is obtained through linear regression. For better fitting result, K_a should be tried over a large range to obtain the most accurate value.

<1> High concentration induced aggregation

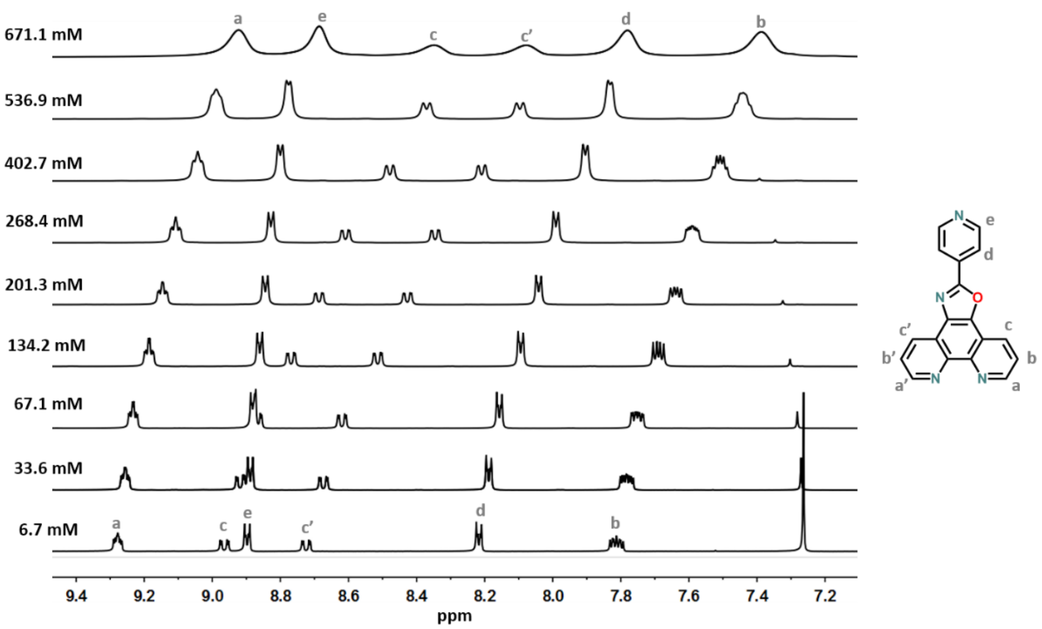

Supplementary Fig. 17. Stacked ^{1}H NMR spectra of **C10** in $CDCl_3$ with gradually increased concentration.

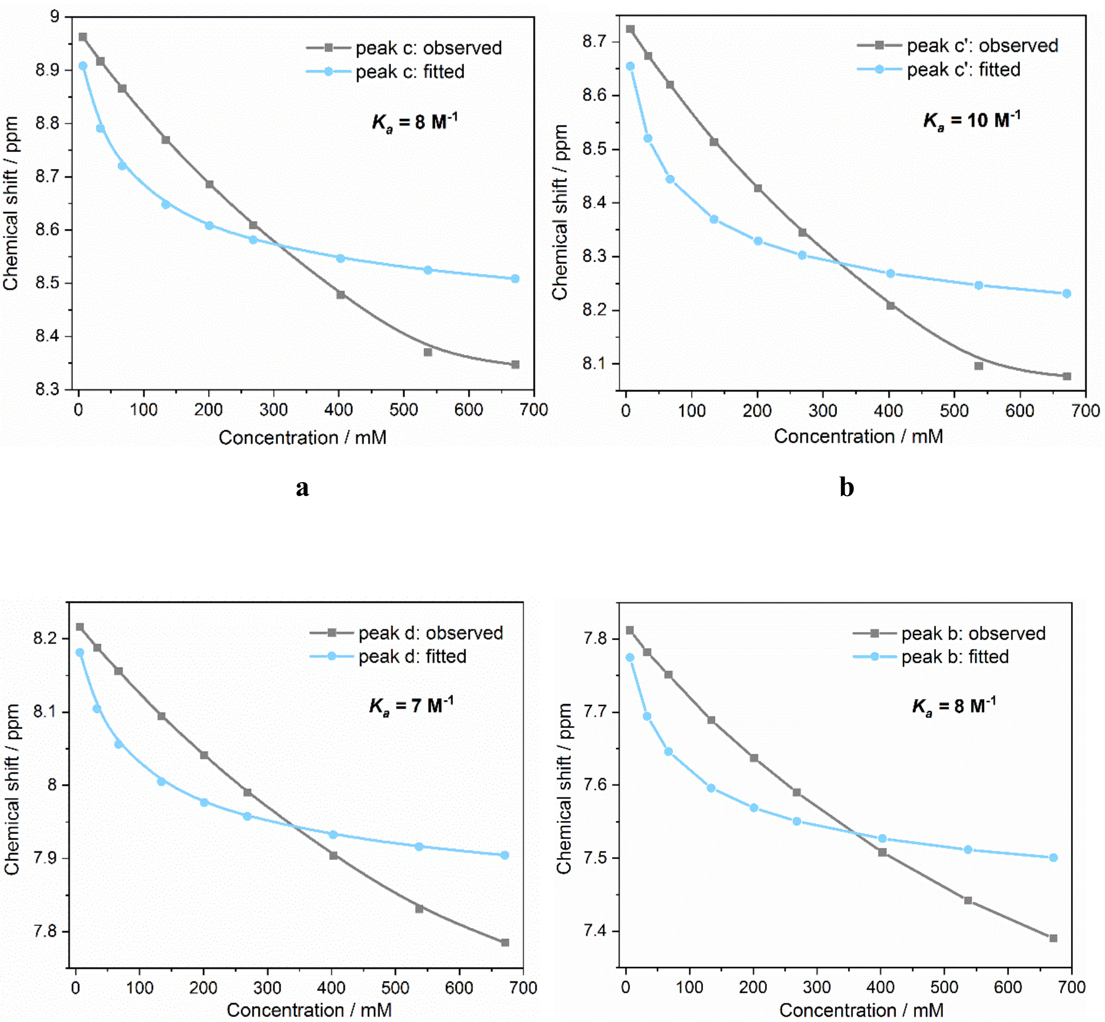

c **d**

Supplementary Fig. 18. The chemical shifts of **C10** against concentrations in $CDCl_3$ and their corresponding non-linear fitted curves to give K_a (a-d). (***Note***: it should be pointed out that the fitting method gives relatively unsatisfied results especially in high concentration region, which means there exist other oligomers beside the monomer and dimer.)

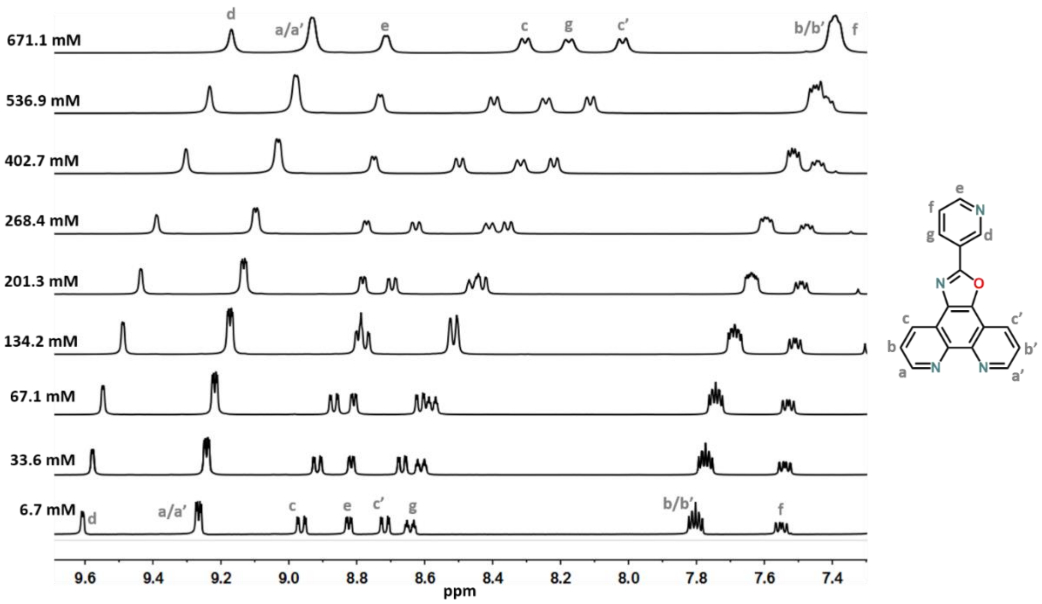

Supplementary Fig. 19. Stacked ^{1}H NMR spectra of **C11** in $CDCl_3$ with gradually increased concentration.

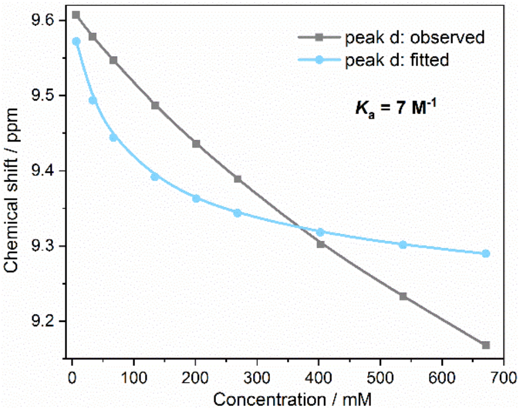

a

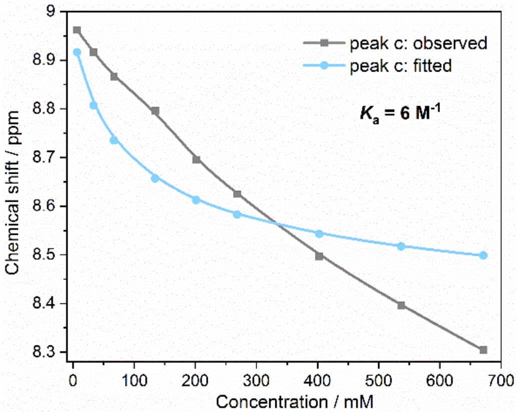

b

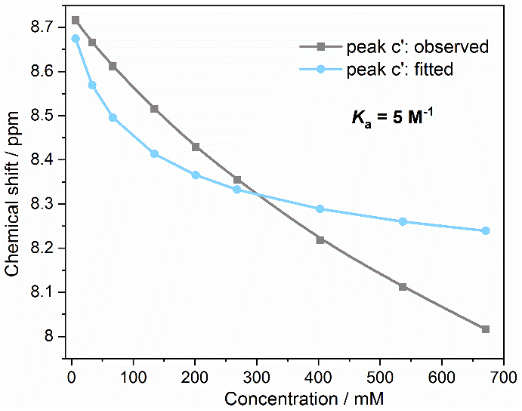

c

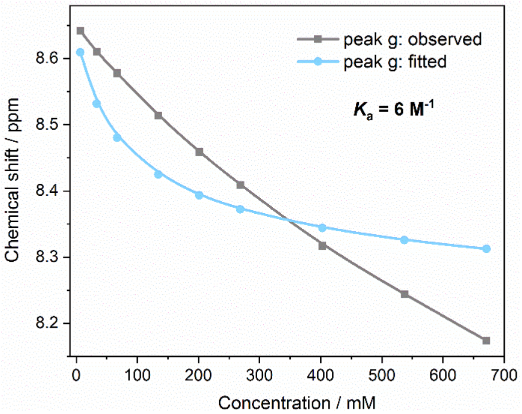

d

Supplementary Fig. 20. The chemical shifts of **C11** against concentrations in $CDCl_3$ and their corresponding non-linear fitted curves to give K_a (a-d).

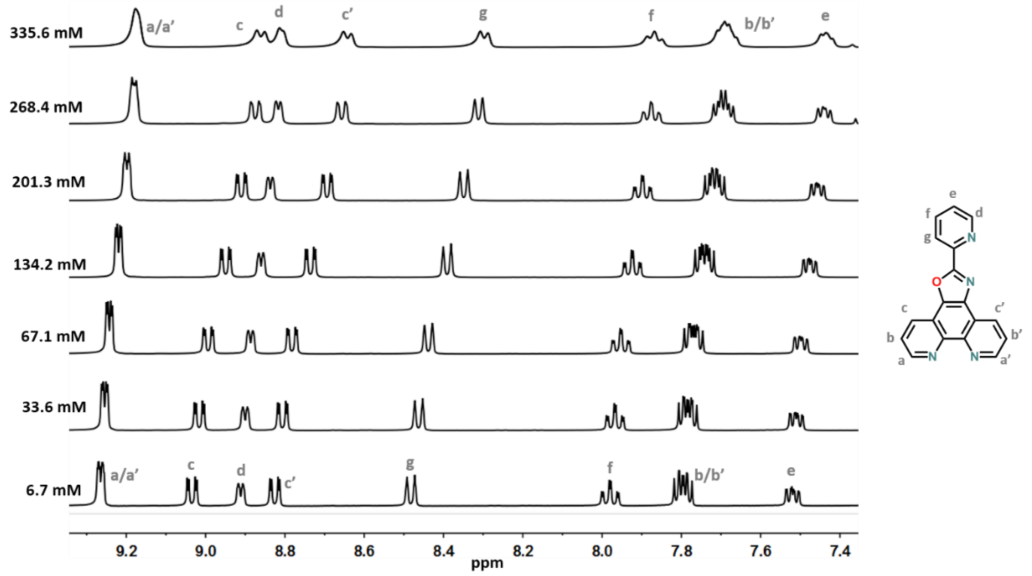

Supplementary Fig. 21. Stacked ^{1}H NMR spectra of **C12** in $CDCl_3$ with gradually increased concentration.

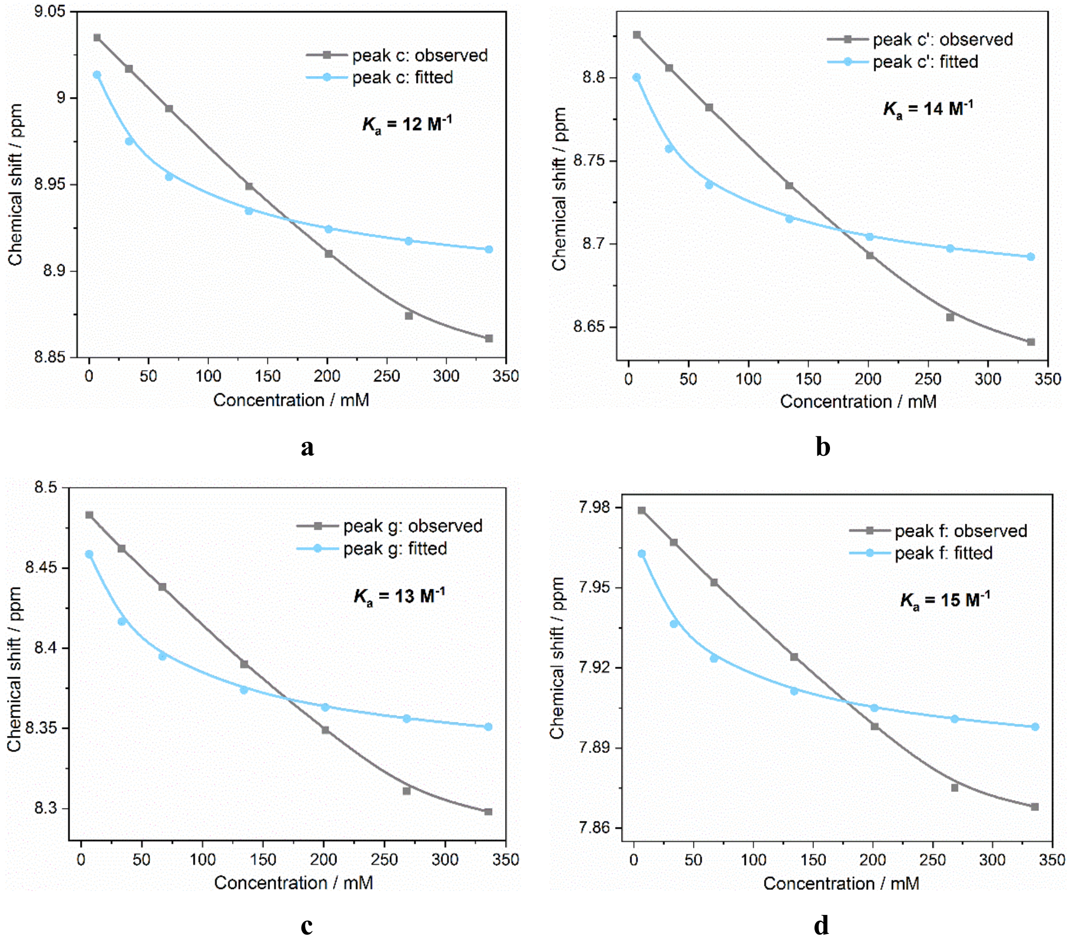

Supplementary Fig. 22. The chemical shifts of **C12** against concentrations in $CDCl_3$ and their corresponding non-linear fitted curves to give K_a (a-d).

<2> Variable solvent ratios induced aggregation

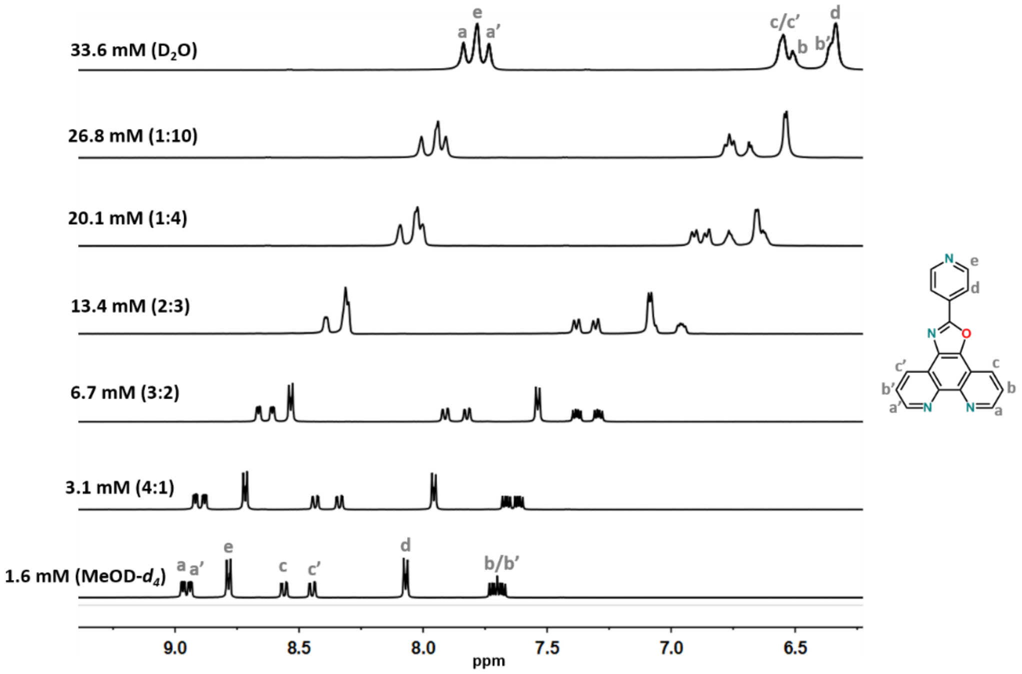

Supplementary Fig. 23. Stacked ^{1}H NMR spectra of **C10** in MeOD-d_4/D_2O mixture with gradually increased concentration. (***Note***: **C10** in D_2O with high concentration was prepared as the starting sample, then diluted with MeOD-d_4 to certain volume ratio to obtain its corresponding ^{1}H NMR spectrum.)

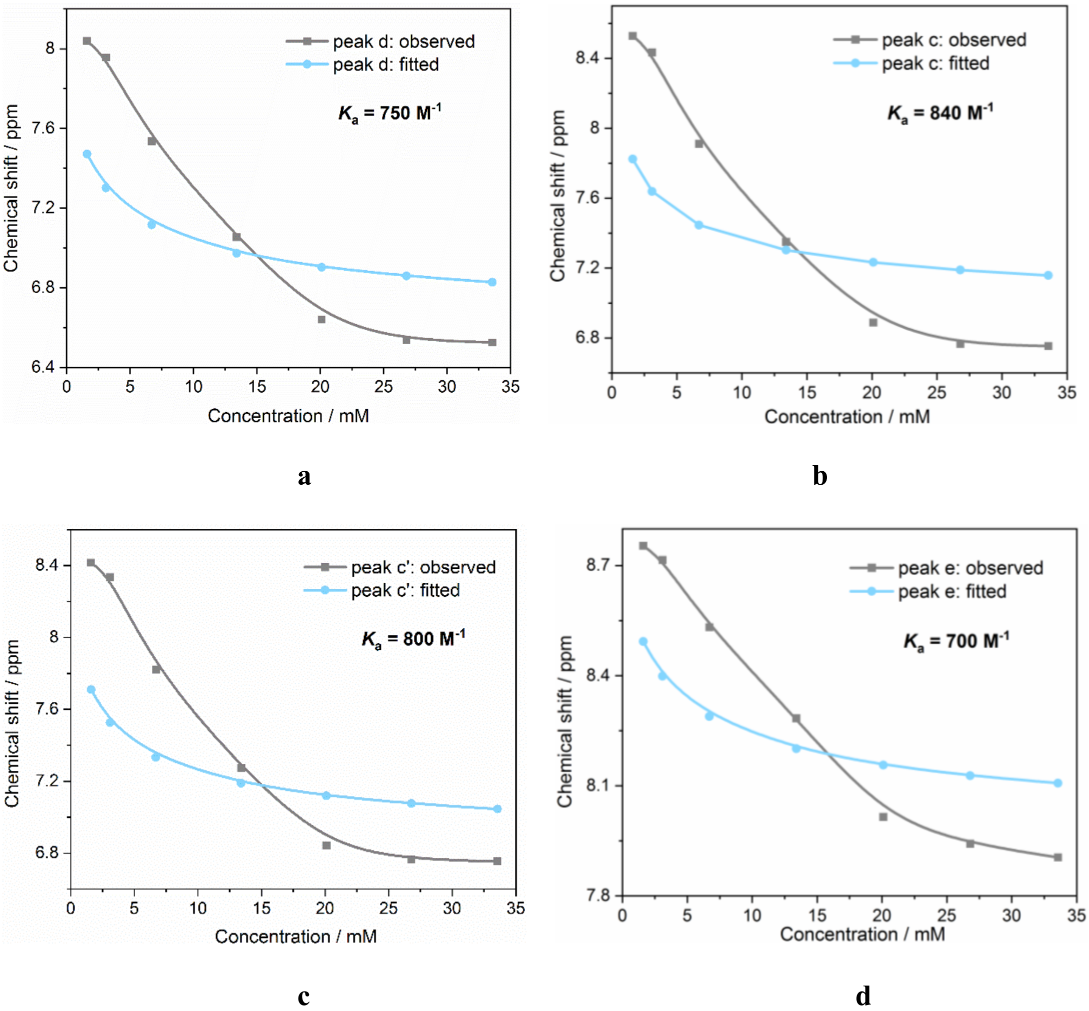

Supplementary Fig. 24. The chemical shifts of **C10** against concentrations in solvents mixture and their corresponding non-linear fitted curves to give K_a (a-d). (***Note***: the deviations between experimental and fitted data indicate that there exist other oligomers beside the monomer and dimer, which is consistent with other results. So, K_a represents the apparent association constant rather than that for the dimer.)

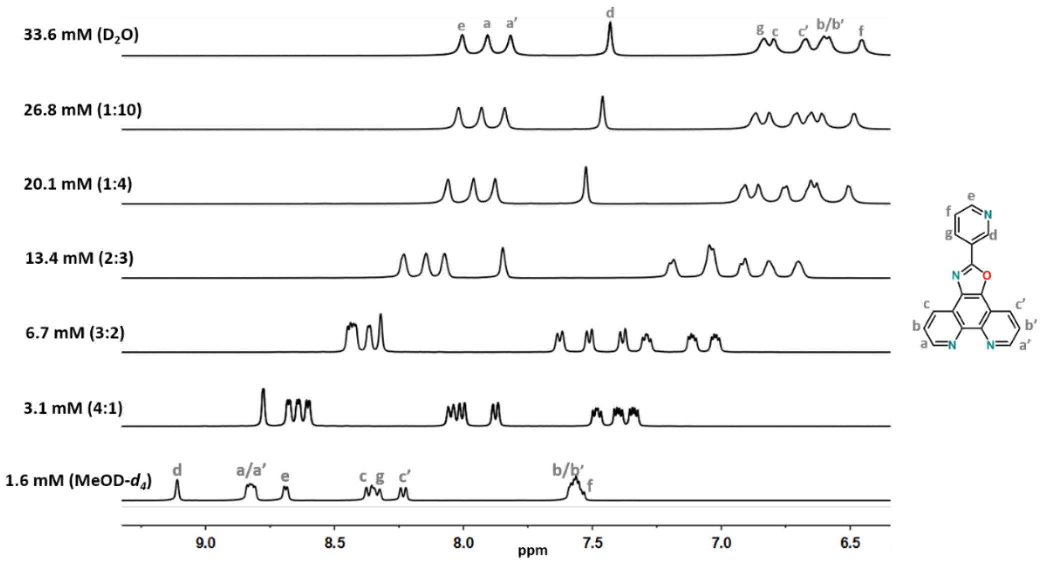

Supplementary Fig. 25. Stacked ^{1}H NMR spectra of **C11** in MeOD-d_4/D_2O mixture with gradually increased concentration.

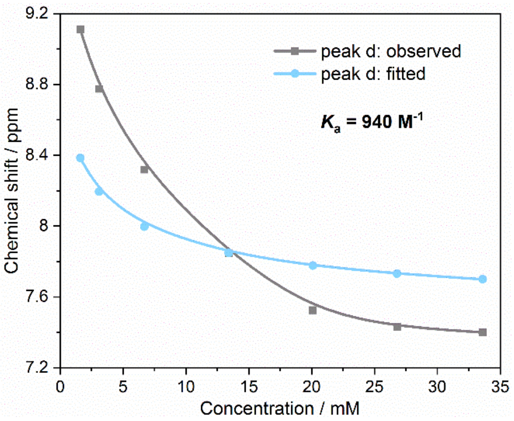

a

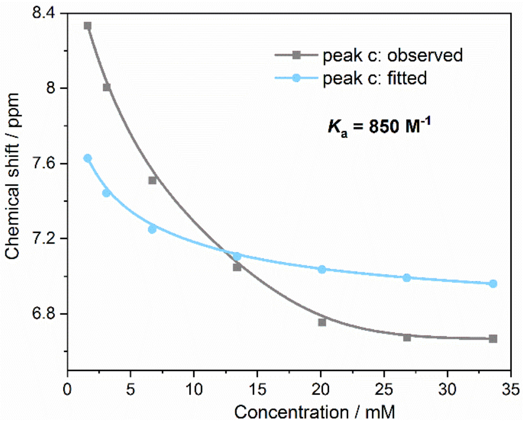

b

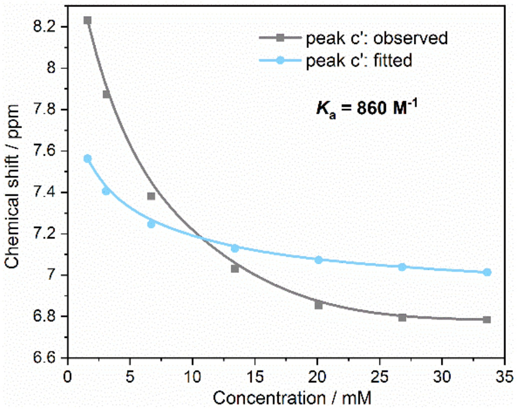

c

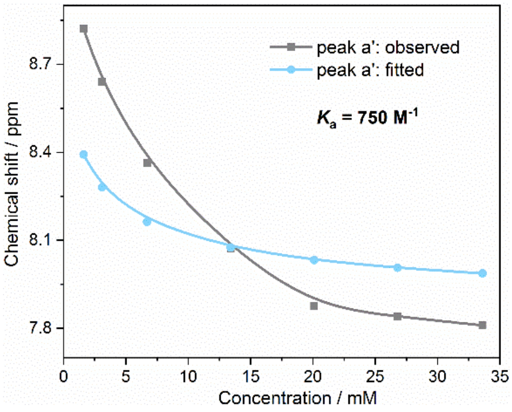

d

Supplementary Fig. 26. The chemical shifts of **C11** against concentrations in solvents mixture and their corresponding non-linear fitted curves to give K_a (a-d).

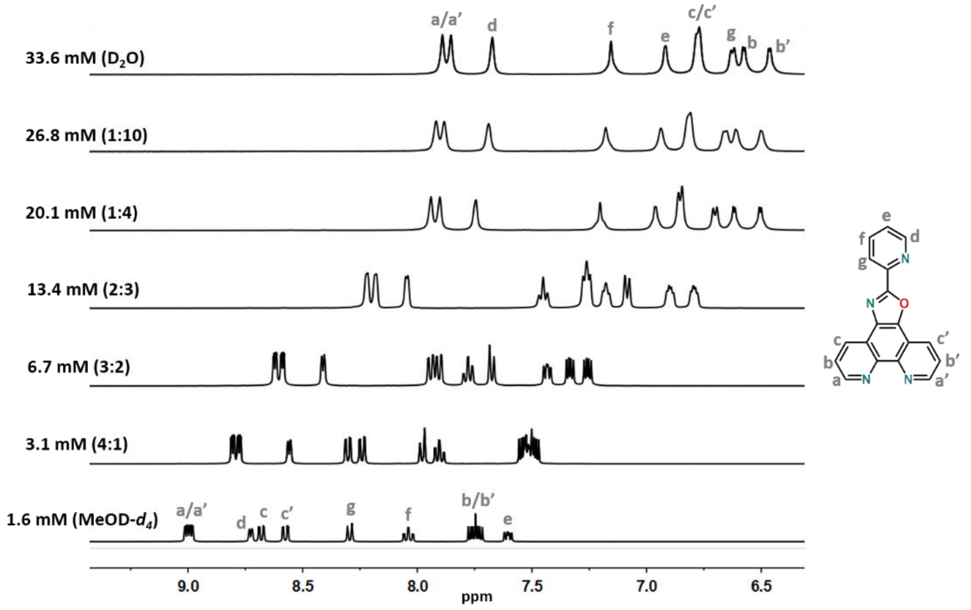

Supplementary Fig. 27. Stacked ^{1}H NMR spectra of **C12** in MeOD-d_4/D_2O mixture with gradually increased concentration.

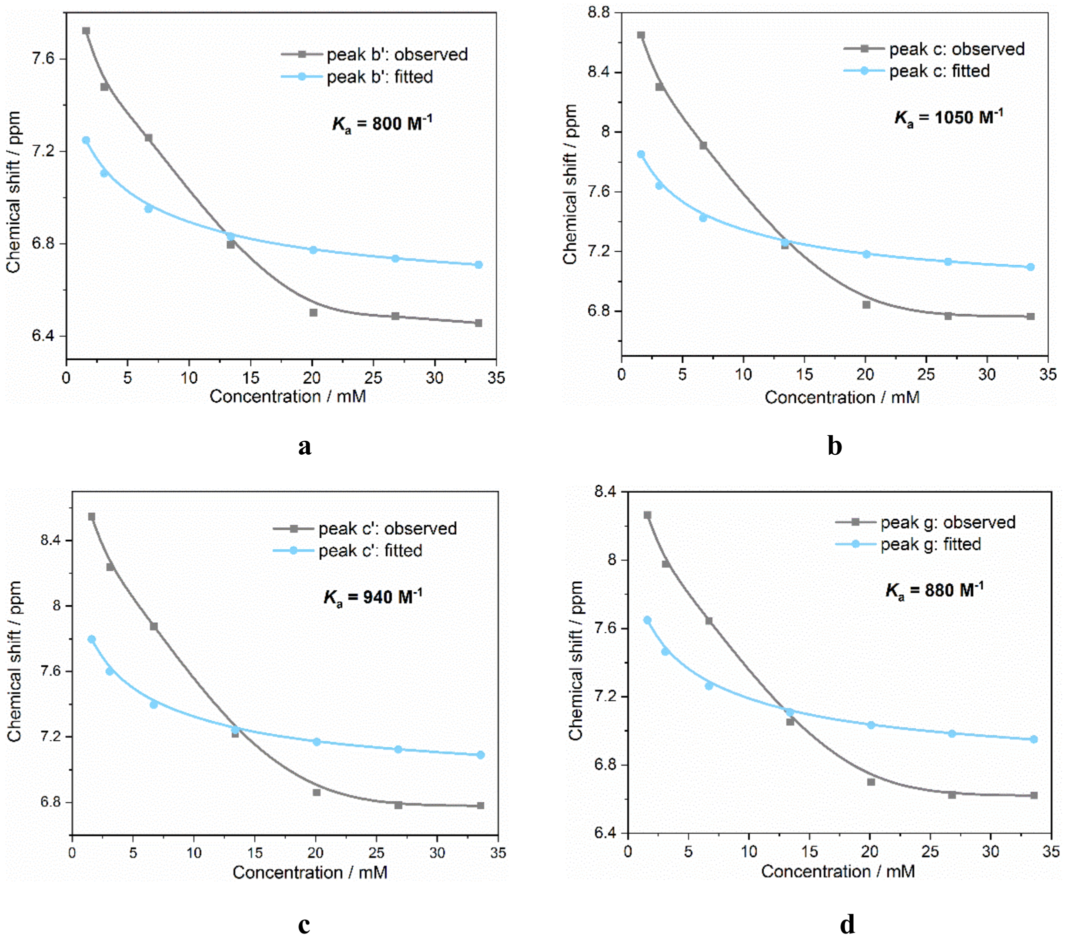

Supplementary Fig. 28. The chemical shifts of **C12** against concentrations in solvents mixture and their corresponding non-linear fitted curves to give K_a (a-d).

2.2 AFM measurements

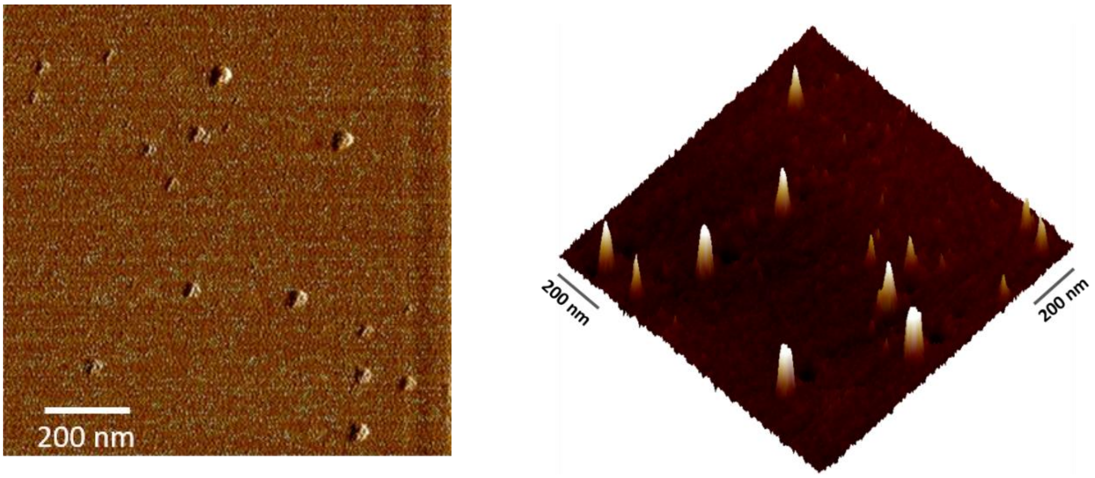

a **b**

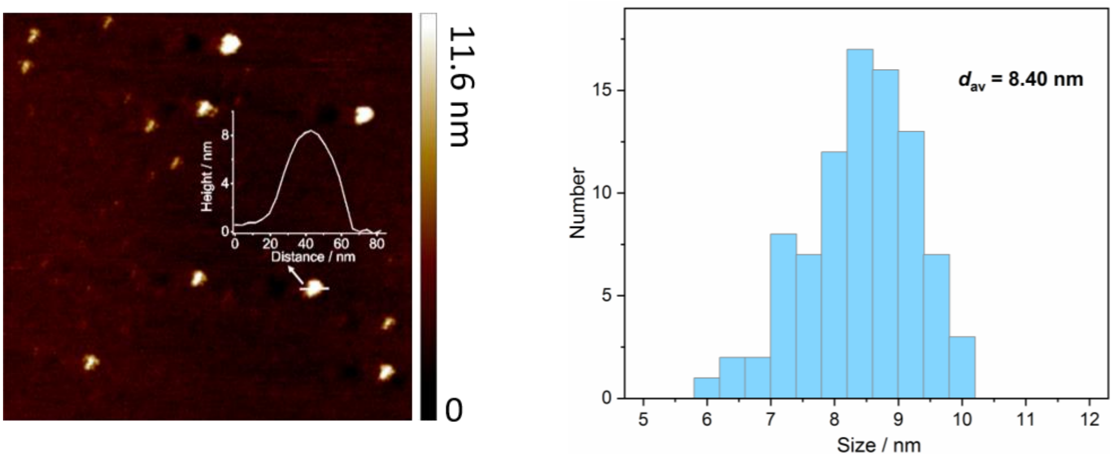

c **d**

Supplementary Fig. 29. AFM analysis of **C10**-based nanoparticle in wet condition. (a) Pristine AFM image of a solution of **C10** in H_2O (1.5 mM) on mica. (b – c) 3D and 2D height profile of the corresponding area. (d) Statistical distribution of nanoparticle number *versus* size.

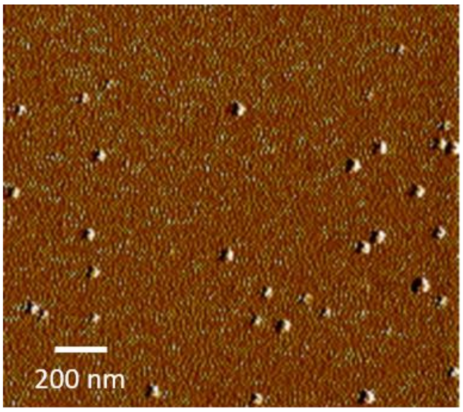

a

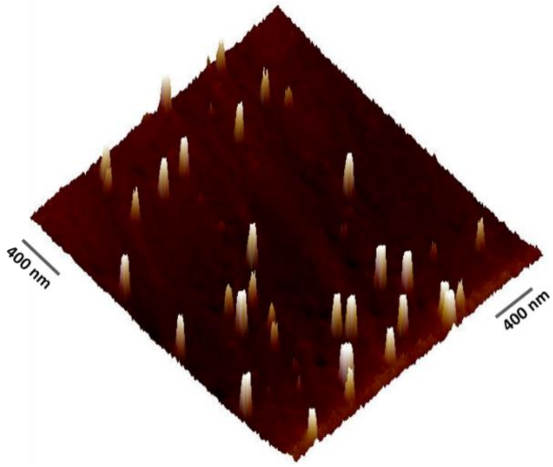

b

Supplementary Fig. 30. AFM analysis of **C11**-based nanoparticle in wet condition. (a) Pristine AFM image of a solution of **C10** in H_2O (1.5 mM) on mica. (b) 3D height profile of the corresponding area.

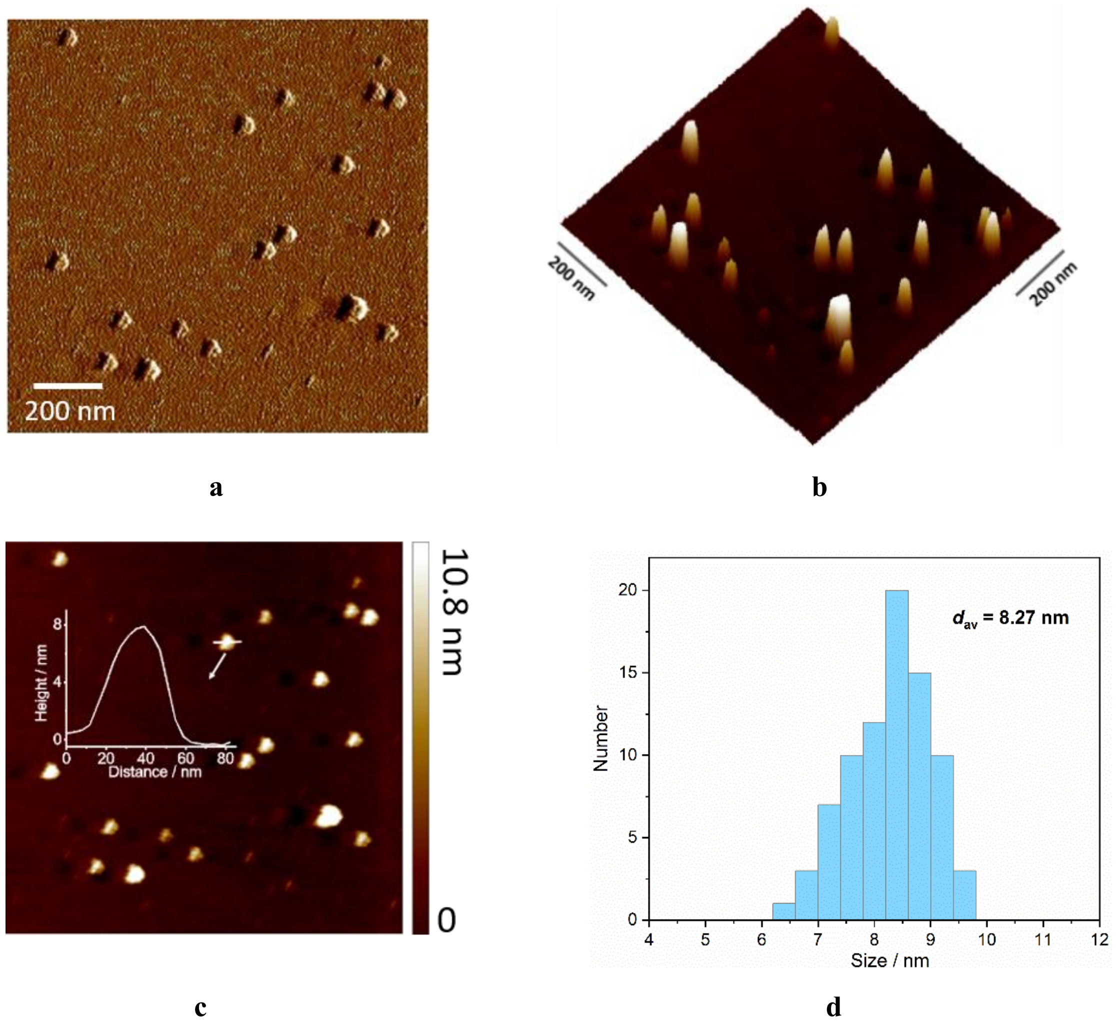

Supplementary Fig. 31. AFM analysis of **C12**-based nanoparticle in wet condition. (a) Pristine AFM image of a solution of **C12** in H_2O (1.5 mM) on mica. (b – c) 3D and 2D height profile of the corresponding area. (d) Statistical distribution of nanoparticle number *versus* size.

2.3 Single crystal X-ray analysis

These structures were solved by direct method and refined by full-matrix least squares against F^2 using the *olex2-1.3* software.[5] For **C10** and **C12**, the hydrogen atoms were placed in geometrically calculated positions and included in the refinement process using riding model. For **C11**, the hydrogen atoms were found through the difference of electron density map and refined isotropically. Crystal and refinement parameters are listed in Table S4.

Supplementary Table 2. Crystal data and structure refinement results

**Identification code**	**C10**	**C11**	**C12**
Empirical formula	$C_{18}H_{10}N_4O$	$C_{18}H_{10}N_4O$•H_2O	$C_{18}H_{10}N_4O$•$CHCl_3$
Formula weight	298.30	316.32	417.67
Temperature/K	290	290	290
Crystal system	orthorhombic	monoclinic	orthorhombic
Space group	*P*bca	$P2_1$/c	*P*nma
a/Å	7.2934(2)	9.4007(4)	9.8115(3)
b/Å	17.7064(3)	22.4279(9)	6.7761(2)
c/Å	21.5457(5)	7.2003(3)	27.3181(8)
α/°	90	90	90
β/°	90	108.239(4)	90
γ/°	90	90	90
Volume/$Å^3$	2782.41(11)	1441.83(11)	1816.21(9)
$\rho_{calc,}$ g/cm^3	1.424	1.457	1.527
μ/mm^{-1}	0.754	0.812	4.720
F(000)	1232.0	656.0	848.0
Reflections collected	5812	5420	4405
Independent reflections	2689	2770	1912
Data/restraints/parameters	2689/1/208	2770/0/265	1912/0/160
Goodness-of-fit on F^2	1.083	1.095	1.055
Final R indexes [I>=2σ (I)]	R_1 = 0.0399, wR_2 = 0.1063	R_1 = 0.0459, wR_2 = 0.1465	R_1 = 0.0508, wR_2 = 0.1378
Final R indexes [all data]	R_1 = 0.0480, wR_2 = 0.1127	R_1 = 0.0637, wR_2 = 0.1571	R_1 = 0.0565, wR_2 = 0.1445
Largest diff. peak/hole / e $Å^{-3}$	0.16/-0.21	0.18/-0.24	0.43/-0.58
CCDC Number	2072956	2072957	2072958

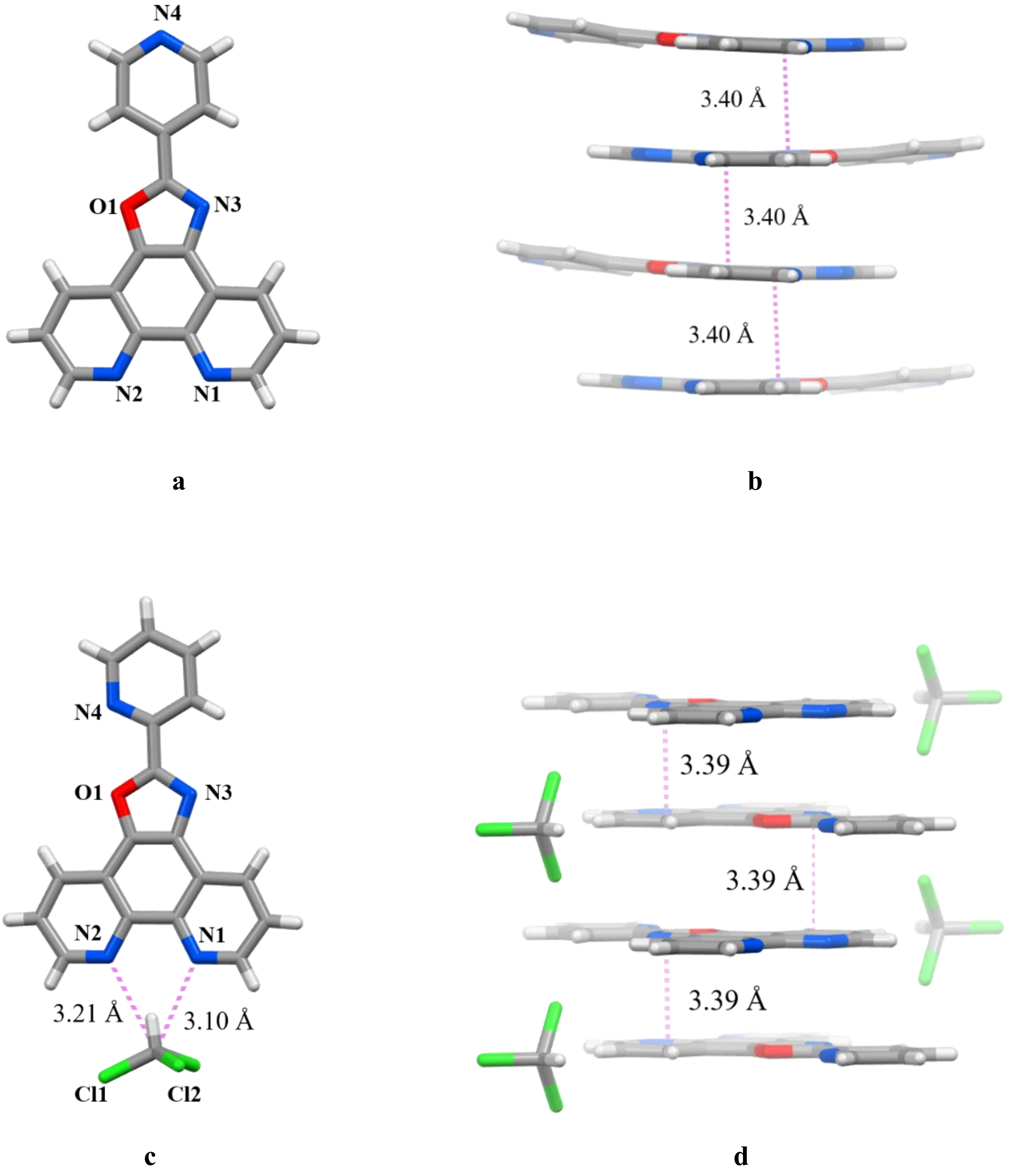

Supplementary Fig. 32. Crystal structure of **C10** and **C12**. (a) molecular structure of **C10**; (b) packing mode of **C10** along *c* axis and the interlayer distances are labeled; (c) molecular structure of **C12** and hydrogen bonding with $CHCl_3$; (d) packing mode of **C12** along *c* axis and the interlayer distances are labeled.

2.4 Luminescent emission

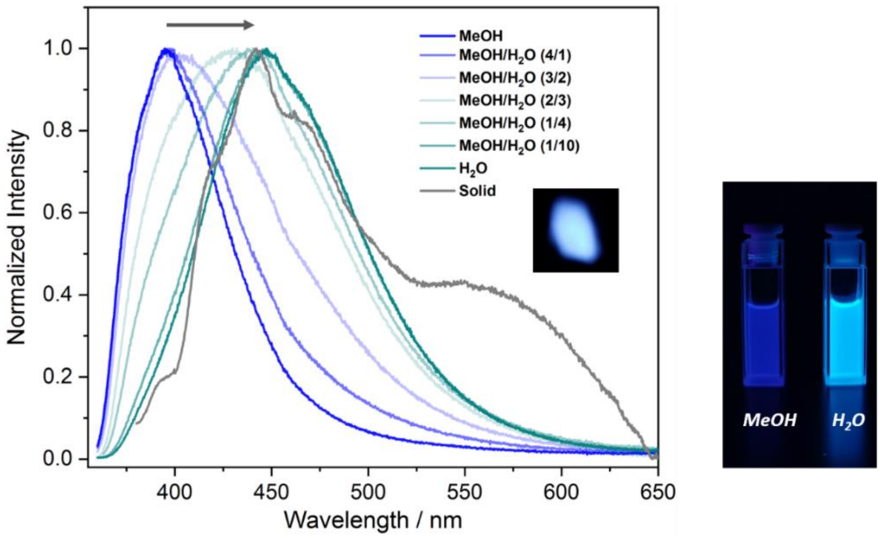

Supplementary Fig. 33. Luminescent emission of **C11** under 365 nm radiation. Left: the emission spectra in liquid with viable solvent ratio and in solid state (inserted is the photograph of solid sample upon excitation). Right: the photograph of emission in methanol and water, respectively.

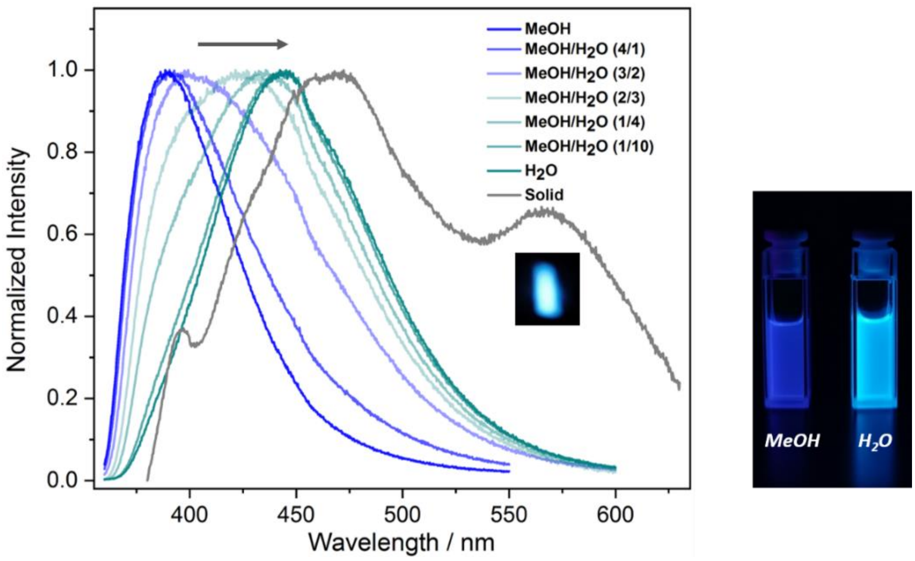

Supplementary Fig. 34. Luminescent emission of **C12** under 365 nm radiation. Left: the emission spectra in liquid with viable solvent ratio and in solid state (inserted is the photograph of solid sample upon excitation). Right: the photograph of emission in methanol and water, respectively.

3. Supplementary References

1. Chen, X. L. *et al.* Assembly of two novel cadmium(II) supramolecular architectures constructed from pyridine-functionalized 1,10-phenanthroline ligand. *Inorganica Chim. Acta* **362**, 3963-3968 (2009).
2. Doroshenko, A. O., Posokhov, E. A., Shershukov, V. M., Mitina, V. G. & A., P. O. Spectral and luminescence properties of derivatives of 2-aryl[9,10]phenanthroxazole. *Chem. Heterocycl. Compd.* **31**, 492-499 (1995).
3. Hansen, C. M. *Hansen solubility parameters : a user's handbook*. 2nd edn, (CRC Press, 2007).
4. Horman, I. & Dreux, B. Estimation of Dimerisation Constants from Complexatin-Induced Displacements of ^{1}H NMR Chemical Shifts: Dimerisation of Caffeine. *Helv. Chim. Acta* **67**, 754-764 (1984).
5. Dolomanov, O. V., Bourhis, L. J., Gildea, R. J., Howard, J. A. K. & Puschmann, H. OLEX2: a complete structure solution, refinement and analysis program. *J. Appl. Cryst.* **42**, 339-341 (2009).
