# Supplementary material for: Precise heteroatom doping determines aqueous solubility and self-assembly behaviors for polycyclic aromatic skeletons
Source: Commun Chem. 2022 Aug 29;5:104. doi: 10.1038/s42004-022-00724-1 (PMC9814590; doi:10.1038/s42004-022-00724-1)
Supplement: Supplementary file 3 — Description of Additional Supplementary Files [file 42004_2022_724_MOESM3_ESM.docx]

Description of Additional Supplementary Files

**File name:** Supplementary Data 1

**Description:** cif file of C10

**File name:** Supplementary Data 2

**Description:** cif file of C11

**File name:** Supplementary Data 3

**Description**: cif file of C12
