# Supplementary material for: Precise heteroatom doping determines aqueous solubility and self-assembly behaviors for polycyclic aromatic skeletons
Source: Commun Chem. 2022 Aug 29;5:104. doi: 10.1038/s42004-022-00724-1 (PMC9814590; doi:10.1038/s42004-022-00724-1)
Supplement: Supplementary file 1 — Li_PR File [file 42004_2022_724_MOESM1_ESM.pdf]

nature portfolio

Peer Review File

Precise heteroatom doping determines aqueous solubility and self-assembly behaviours for polycyclic aromatic skeletons

Open Access This file is licensed under a Creative Commons Attribution 4.0 International License, which permits use, sharing, adaptation, distribution and reproduction in any medium or format, as long as you give appropriate credit to the original author(s) and the source, provide a link to the Creative Commons license, and indicate if changes were made. In the cases where the authors are anonymous, such as is the case for the reports of anonymous peer reviewers, author attribution should be to 'Anonymous Referee' followed by a clear attribution to the source work. The images or other third party material in this file are included in the article's Creative Commons license, unless indicated otherwise in a credit line to the material. If material is not included in the article's Creative Commons license and your intended use is not permitted by statutory regulation or exceeds the permitted use, you will need to obtain permission directly from the copyright holder. To view a copy of this license, visit http://creativecommons.org/licenses/by/4.0/.

Reviewers' comments:

Reviewer #1 (Remarks to the Author):

Review report on "Precise N/O doping determines aqueous solubility and self-assembly behaviors for polycyclic aromatic skeleton"

This manuscript describes intentional control of hydrophilicity of aromatic derivatives by introducing N as well as O into their skeleton, some of which eventually become highly water-soluble. The reviewer believes the subject of precise control of physical properties by appropriately combining building blocks is quite interesting and well along with recent research trend, which is potentially worth to be published in Communications Chemistry. Also, its structural investigations combined with NMR, AFM, and XRD (as well as excited emission spectra) are convincing and well-organized. Moreover, their presentation is well-written, and figures are easy-to-see. However, I found some fatal flaws in their discussion related to Hansen solubility parameters, thus, the flaws should be amended before formal acceptance for publication.
The first apparent flaw is "Which solubility parameters is discussed, of Hansen or Hoy?" Hansen solubility parameters (HSP) is based on cohesive energy density, which is divided into three parameters of dD, dP, and dH. The HSP has been utilized for long period to explain solubility, dispersibility, and compatibility, which contains huge database and intellectual infrastructure, and long history. On the other hand, although Hoy's parameters also consist of three parameters of dD, dP, and dH, its underlying concept is unknown, and Hoy's parameters are deemed (in HSP community) to be essentially different from HSP. For example, Hoy's dD in absolute value is extraordinary smaller than that of HSP; in fact, dD of 6.3 for C10-C12 compounds calculated via Hoy's GC method is one-half of those of fluorocarbon-derivatives (with smallest dD values in HSP). Therefore, HSP and Hoy's parameters should not be compared and mixed in one discussion. Please manifest which solubility parameters is discussed here (I believe only HSP should be discussed in this study).
The second flaw is related to the question, "Which HSP set for water is appropriate for this case?" At least, three sets of HSP for water are known. The first is the HSP of single-molecule (pure) water ([dD, dP, dH] = [15.5, 16, 42.3]), which the authors adopted in this manuscript. This set well explains strong (hydrogen-bonding) H2O-H2O interaction; however, its use in prediction of solubility in water is not deemed as appropriate in most case. Water is unique solvent due to its extremely small molecular size and strongest hydrogen bonding, interaction of which is quite hard to systematically predict even in HSP methodology. Although there is no concrete consensus in the HSP community on how to deal with water, I believe the following procedure would be the best of bad situation. The second and third sets of HSP for water are those derived from solubility data (which set is appropriate should be chosen case-by-case). The second set of HSP for water was derived from experimental dataset of >1%-soluble compounds in water, which may be appropriate for the diluted solute case. The third set of HSP for water was derived from experimental dataset of complete miscible compounds with water, which may be appropriate for the dense solute case. In the present case, I believe both second and third sets for water HSP (not for pure water) should be considered.
I calculated the HSPs of C1-C12 compounds by Y-MB prediction tool in commercial package software HSPiP (version 5.3.08) and compared them with second and third sets of water HSP (see attached excel file), which shows REDs of them were over 1.0; hence, suggesting insoluble in water. I guess the straightforward prediction power of HSP is limited in the case of water solubility and/or large and complex molecules; however, we can go more deeper with both HSP and thoughtful insight. In the case of complex-structure compounds, the HSPs of partial segment of the compounds may be critical. I picked up HSPs (from official database as well as the Y-MB prediction tool, see the attached excel file) of the partial segment (building blocks of C1-C12), and compared with water HSPs, which shows REDs for some of building blocks are <1.0; hence, potentially soluble in water. The building blocks of C10-C12 with the largest RED for water were "Bipyridine" and "Phenanthroline" with REDs of 1.1-1.3, suggesting insoluble in water. In other words, if this "Bipyridine" and/or "Phenanthroline" parts were effectively screened out from water exposure, the C10-C12 compounds should be soluble in water. In fact, the authors' investigation suggested C10-C12 solutes aggregated as nano particles in water

through pi-pi stacking. If the authors can propose appropriate stacking configuration to screen out "Bipyridine" and/or "Phenanthroline" parts (more precisely, to screen out aromatic carbons (not aromatic nitrogens and oxygens) in these parts) with help from structural information via NMR and XRD, the solubility of C10-C12 in water are to be well explained in HSP framework. Please consider reconstructing your discussion along with above-mentioned scenario or other more appropriate ones.

Daisuke Nakamura

Reviewer #2 (Remarks to the Author):

The manuscript by Li et al. describes a concept to enhance the aqueous solubility of polycyclic aromatic hydrocarbons (PAHs). As PAHs are important class of molecules in many applications, it would be indeed important to establish molecular design concept regarding aqueous solubility. However, what was demonstrated herein is reasonably expected without any surprise; introducing N and O atoms within the PAH cores should polarizes the molecules and enhance the solubility in polar solvents. Though the authors highlight the concept as precise N/O doping, in my opinion, any organic synthesis-based materials-design is precise. Experiments were thoroughly conducted, but I do not find a new finding which attract the broad readership of Communications Chemistry. I recommend this manuscript to other journals such as Scientific Reports.

One comment: I am concerned whether hydrophilicity and water solubility can be separately discussed. If molecules are highly soluble in water, it would be difficult to define the water contact angle as the molecules will be dissolved during the measurement of water-contact angle.

Dear Editor and Reviewers:

We thank you kindly for the very thoughtful and helpful feedback on our manuscript entitled "Precise N/O doping determines aqueous solubility and self-assembly behaviors for polycyclic aromatic skeleton" for potential publication on *Communications Chemistry*. Our response to the constructive suggestions is listed below in blue point by point.

Reviewers' comments:

Reviewer #1 (Remarks to the Author):

Comment: *"This manuscript describes intentional control of hydrophilicity of aromatic derivatives by introducing N as well as O into their skeleton, some of which eventually become highly water-soluble. The reviewer believes the subject of precise control of physical properties by appropriately combining building blocks is quite interesting and well along with recent research trend, which is potentially worth to be published in Communications Chemistry. Also, its structural investigations combined with NMR, AFM, and XRD (as well as excited emission spectra) are convincing and well-organized. Moreover, their presentation is well-written, and figures are easy-to-see. However, I found some fatal flaws in their discussion related to Hansen solubility parameters, thus, the flaws should be amended before formal acceptance for publication."*

Response: We thank the reviewer for the recognition of our work and supportive feedback, and address their concerns below.

Comment: *"The first apparent flaw is "Which solubility parameters is discussed, of Hansen or Hoy?" Hansen solubility parameters (HSP) is based on cohesive energy density, which is divided into three parameters of dD, dP, and dH. The HSP has been utilized for long period to explain solubility, dispersibility, and compatibility, which contains huge database and intellectual infrastructure, and long history. On the other hand, although Hoy's parameters also consist of three parameters of dD, dP, and dH, its underlying concept is unknown, and Hoy's parameters are deemed (in HSP community) to be essentially different from HSP. For example, Hoy's dD in absolute value is extraordinary smaller than that of HSP; in fact, dD of 6.3 for C10-C12 compounds calculated via Hoy's GC method is one-half of those of fluorocarbon-derivatives (with smallest dD values in HSP). Therefore, HSP and Hoy's parameters should not be compared and mixed in one discussion. Please manifest which solubility parameters is discussed here (I believe only HSP should be discussed in this study)."*

Response: We thank the reviewer for the professional advice. At the beginning, due to the complexity and experimentally unmeasurable nature of the HSP of these nanoparticles, we tried to find a given model to directly calculate these parameters, thus resulting in the mixed and unproper use of the two solubility parameters simultaneously. Now, the calculations, discussions and references related to Hoy's parameters are all removed from the article and supporting information. The Hansen solubility parameters as the only solubility parameters are

used across the whole paper.

Comment: *"The second flaw is related to the question, "Which HSP set for water is appropriate for this case?" At least, three sets of HSP for water are known. The first is the HSP of single-molecule (pure) water ([dD, dP, dH] = [15.5, 16, 42.3]), which the authors adopted in this manuscript. This set well explains strong (hydrogen-bonding) H_2O-H_2O interaction; however, its use in prediction of solubility in water is not deemed as appropriate in most case. Water is unique solvent due to its extremely small molecular size and strongest hydrogen bonding, interaction of which is quite hard to systematically predict even in HSP methodology. Although there is no concrete consensus in the HSP community on how to deal with water, I believe the following procedure would be the best of bad situation. The second and third sets of HSP for water are those derived from solubility data (which set is appropriate should be chosen case-by-case). The second set of HSP for water was derived from experimental dataset of >1%-soluble compounds in water, which may be appropriate for the diluted solute case. The third set of HSP for water was derived from experimental dataset of complete miscible compounds with water, which may be appropriate for the dense solute case. In the present case, I believe both second and third sets for water HSP (not for pure water) should be considered."*

Response: We thank the reviewer for pointing out this issue, which is crucial for the global HSP analysis and discussion. Following the reviewer's suggestion, we have deleted all the first water HSP set-based calculations and added the second and third water HSP set-based calculations in Table 1 that are highlighted by yellow. For comparison, both water HSP were used for C1-C12 molecules. Unexpectedly, the selective use of the second and third Water HSP set based on different conditions could give a satisfying prediction and explanation of the hydrophilicity and aqueous solubility performance of C1-C12, especially for both N and O doping series C7-C12 (C7-C9 for dilute solute condition, C10-C12 for dense solute condition). Typically, using the third set of water HSP for C10-C12 gave their REDs ≤1.0, which is consistent with the experimental results and seems no need to find other methods for HSP calculations like the unproperly used Hoy's method. Thus, we have deleted the previous first set of water HSP-based discussions and reconstructed the discussion based on the more reasonable water HSP set. These changes are highlighted by yellow on Pages 7-8.

Comment: *"I calculated the HSPs of C1-C12 compounds by Y-MB prediction tool in commercial package software HSPiP (version 5.3.08) and compared them with second and third sets of water HSP (see attached excel file), which shows REDs of them were over 1.0; hence, suggesting insoluble in water. I guess the straightforward prediction power of HSP is limited in the case of water solubility and/or large and complex molecules; however, we can go more deeper with both HSP and thoughtful insight. In the case of complex-structure compounds, the HSPs of partial segment of the compounds may be critical. I picked up HSPs (from official database as well as the Y-MB prediction tool, see the attached excel file) of the partial segment (building blocks of C1-C12), and compared with water HSPs, which shows REDs for some of building blocks are <1.0; hence, potentially soluble in water. The building blocks of C10-C12 with the largest RED for water were "Bipyridine" and "Phenanthroline" with REDs of 1.1-1.3, suggesting insoluble in water. In other words, if this "Bipyridine" and/or "Phenanthroline"*

parts were effectively screened out from water exposure, the C10-C12 compounds should be soluble in water. In fact, the authors' investigation suggested C10-C12 solutes aggregated as nano particles in water through pi-pi stacking. If the authors can propose appropriate stacking configuration to screen out "Bipyridine" and/or "Phenanthroline" parts (more precisely, to screen out aromatic carbons (not aromatic nitrogens and oxygens) in these parts) with help from structural information via NMR and XRD, the solubility of C10-C12 in water are to be well explained in HSP framework. Please consider reconstructing your discussion along with above-mentioned scenario or other more appropriate ones."

Response: We thank the reviewer for the scrupulous calculation by yourself and valuable suggestions. Compared with the Y-MB prediction method, the optimized HSP obtained from a library of organic solvents' evaluation may be more reliable from an experimental point of view. Moreover, following the reviewer's suggestion of choosing a suitable water HSP set, the derived calculations and predictions match well with the experimental results in general, especially for C10-C12 with good to excellent aqueous solubility. Although the REDs of C10-C12 are near the boundary condition, it's assumed that this is the key step to bringing these building blocks to aqueous phase and thus form nanoparticles as thermodynamic ensembles. The detailed discussion about this part was added on page 9 and highlighted with yellow.

For the accurate stacking configuration of these nanoparticles, by far, we could only estimate the sizes by DOSY and AFM testing and deduce the adjacent layer distance and layer number by 2D NMR analysis. Although the single crystal X-ray analysis gave visual structural information, the infinite periodic structure in solid state may be different from the discrete nanoparticle as dissolved state in solution. So, it's hard for us to propose a more advanced stacking configuration to screen out "Bipyridine and/or phenanthroline" parts. The detailed discussion about this part was added on pages 10-11 and highlighted with yellow.

Reviewer #2 (Remarks to the Author):

Comment: *"The manuscript by Li et al. describes a concept to enhance the aqueous solubility of polycyclic aromatic hydrocarbons (PAHs). As PAHs are important class of molecules in many applications, it would be indeed important to establish molecular design concept regarding aqueous solubility."*

Response: We thank the reviewer for the positive comments about our work.

Comment: *"However, what was demonstrated herein is reasonably expected without any surprise; introducing N and O atoms within the PAH cores should polarizes the molecules and enhance the solubility in polar solvents."*

Response: We thank the reviewer for raising the molecular polar issue. As the reviewer mentioned, polarized molecules seem to dissolve in polar solvents, which is in accordance with the notion of like dissolves like and is workable in many cases. However, solubility is a complex matter and the prediction is really challenging especially in water condition. Although comparing the polarity between solutes and solvents can give some hints about their

compatibility, this is rather a qualitative and semiempirical way for complex system. As shown in Table R1, we have calculated the dipole moment of C1-C12 and compared them with their hydrophilicity and aqueous solubility performance. Among these, C6 with the largest dipole moment yet shows negligible aqueous solubility; by contrast, C10 with the second smallest dipole moment even shows excellent aqueous solubility; C12 with a moderate dipole moment holds the best aqueous solubility performance. These observations prove that the polarity rule has failed to predict the solubility issue in our system. In this work, we tried to adopt a more comprehensive and quantitative method (HSP analysis) to figure out the relationship between N/O doping and aqueous solubility. The formation of nanoparticles in water is really beyond expectation. Until now, even though the real HSP of these nanoparticles cannot be directly obtained, we still believe that this is the key step to result in good to excellent aqueous solubility performance for C10-C12, considering the REDs of their molecular-level state are near the boundary condition.

Table R1. Hydrophilicity and aqueous solubility of **C1-C12** and their corresponding dipole moment

	H_2O	C1	C2	C3	C4	C5	C6	C7	C8	C9	C10	C11	C12
Water contact Angle (°)	–	>140	140.6	0	0	0	0	135.2	128.6	0	0	0	0
Aqueous solubility (mg/mL)	–	<0.1	<0.1	<0.1	<0.1	<0.1	<0.1	< 0.1	< 0.1	0.9	40.0	13.0	150.0
Dipole Moment[a] (D)	1.85	0.82	4.73	8.30	5.39	8.94	10.70	1.58	4.36	5.68	1.50	5.12	7.40

a: the calculations were carried out on the Gaussian 09w program. Initially, molecular structure optimizations were conducted with the ground state DFT (B3LYP) method using 6-31G as the basis set. Then, the optimized structures were subjected to the next run for energy calculation.

Comment: *"Though the authors highlight the concept as precise N/O doping, in my opinion, any organic synthesis-based materials-design is precise. Experiments were thoroughly conducted, but I do not find a new finding which attract the broad readership of Communications Chemistry. I recommend this manuscript to other journals such as Scientific Reports."*

Response: We thank the reviewer for raising concern about the term "precise". For sure, any molecular-based synthesis is a precise synthesis. For an individual molecule, there is no need to emphasize its structure obtained from precise design and/or precise synthesis. However, we have designed and synthesized 11 analogous molecules with various N/O doping, each molecule was designed for its specific purpose. The whole doping is from less to more, from simple to complex and covers as many as possible ways to enrich the doping library, which totally shows controllability and intentionality. At this point, it may be suitable for us to describe the N/O doping process with this term.

Comment: *"One comment: I am concerned whether hydrophilicity and water solubility can be separately discussed. If molecules are highly soluble in water, it would be difficult to define the*

water contact angle as the molecules will be dissolved during the measurement of water-contact angle."

Response: We thank the reviewer for raising this question. In our case, the dissolving of C10-C12 in water is relatively a slow process that often needs hand shaking or stirring. When the liquid contacts the solid, it would wet the surface first, then may occur the dissolution process. In practical, when the droplet of water contacts with the pressed surface of C10-C12, it spread around on the interface instantly observed by the digital camera, and thus we believe the water contact angle of 0° is reasonable and reliable data.

Many thanks for your time and consideration.

Kang Li

Senior Scientist
School of Chemistry
South China Normal University

REVIEWERS' COMMENTS:

Reviewer #1 (Remarks to the Author):

I'd like to thank to the authors for revising their manuscript with careful reconsideration. I admit the present discussion of revised manuscript is now concise and appropriate to explain the aqueous solubility phenomenon of their compounds, and recommend to be accepted for the publication in the communications chemistry.

Daisuke Nakamura
